# Supplementary figures and images for: The dorsal fan-shaped body is a neurochemically heterogeneous sleep-regulating center in Drosophila
Source: PLoS Biol. 2025 Mar 26;23(3):e3003014. doi: 10.1371/journal.pbio.3003014 (PMC12135941; doi:10.1371/journal.pbio.3003014)

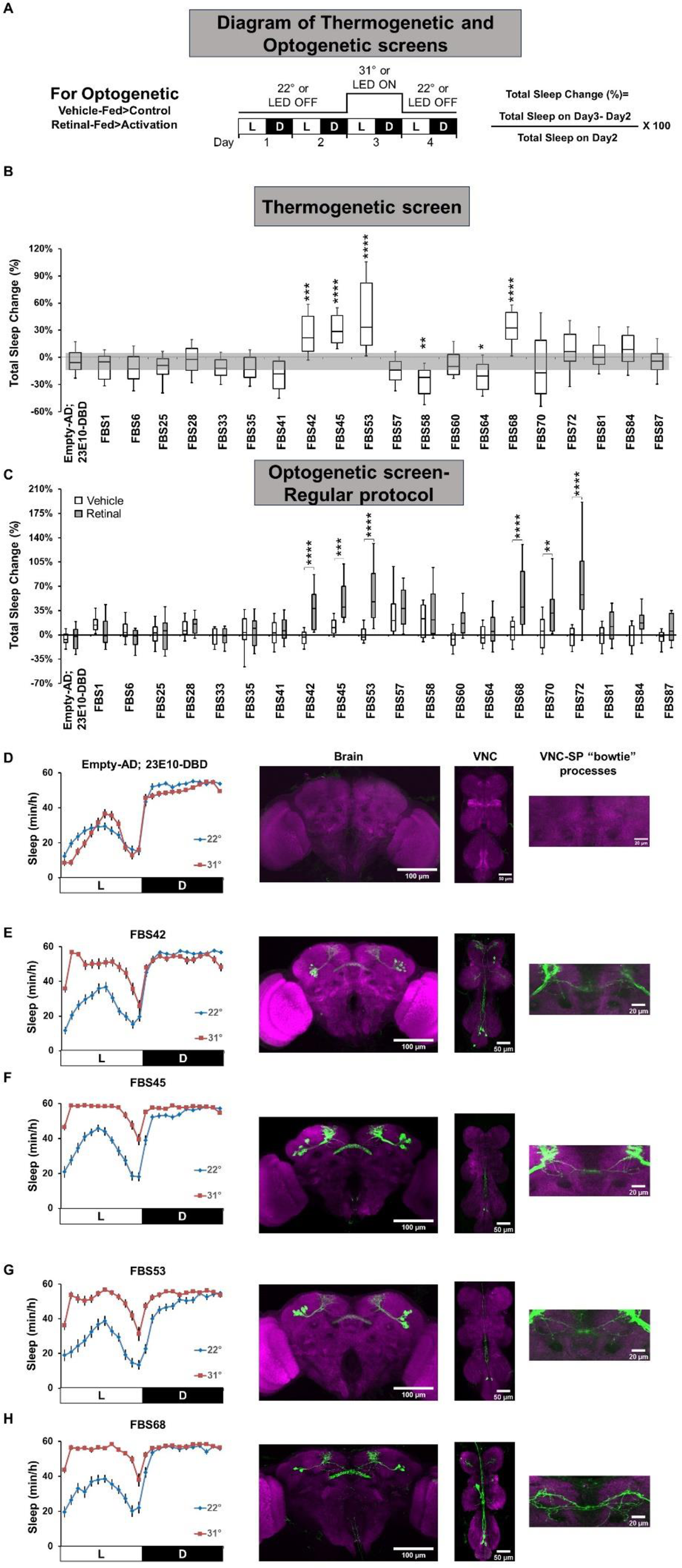

Supplement: S1 Fig — (A) Diagram of the experimental assay. Sleep was measured at 22 °C (for thermogenetic activation) or with LED OFF (for the optogenetic activation protocol) for 2 days to establish baseline sleep profile. Flies were then shifted to 31 °C (thermogenetic) or LEDs were turned ON (LED ON, optogenetic) for 24 h at the start of day 3 to increase activity of the targeted cells by activating the TrpA1 or CsChrimson channel, and then returned to 22 °C or LED OFF on day 4. White bars (L) represent the 12 h of light and black bars (D) represent the 12 h of dark that are oscillating daily. (B) Box plots of total sleep change in % ((total sleep on day 3-total sleep on day 2/total sleep on day 2) × 100) for female control (Empty: Empty-AD; 23E10-DBD) and 20 FBS lines expressing UAS-TrpA1; UAS-mCD8GFP. The bottom and top of each box represents the first and third quartile, and the horizontal line dividing the box is the median. The whiskers represent the 10th and 90th percentiles. The gray rectangle spanning the horizontal axis indicates the interquartile range of the control. Kruskal–Wallis ANOVA followed by Dunn’s multiple comparisons revealed that 4 FBS lines increase sleep significantly more than control flies when thermogenetically activated. *P < 0.05, **P < 0.01, ***P < 0.001, ****P < 0.0001, n = 30–45 flies per genotype. (C) Box plots of total sleep change in % ((total sleep on day 3-total sleep on day 2/total sleep on day 2) × 100) for vehicle-fed and retinal-fed control (Empty) and 20 FBS female flies expressing CsChrimson upon 627 nm LED stimulation. The bottom and top of each box represents the first and third quartile, and the horizontal line dividing the box is the median. The whiskers represent the 10th and 90th percentiles. Two-way ANOVA followed by Sidak’s multiple comparisons revealed that 6 retinal-fed FBS lines increase sleep significantly when stimulated with 627 nm LEDs when compared with vehicle-fed flies. **P < 0.01, ***P < 0.001, ****P < 0.0001, n = 20–40 f [file pbio.3003014.s001.tif]

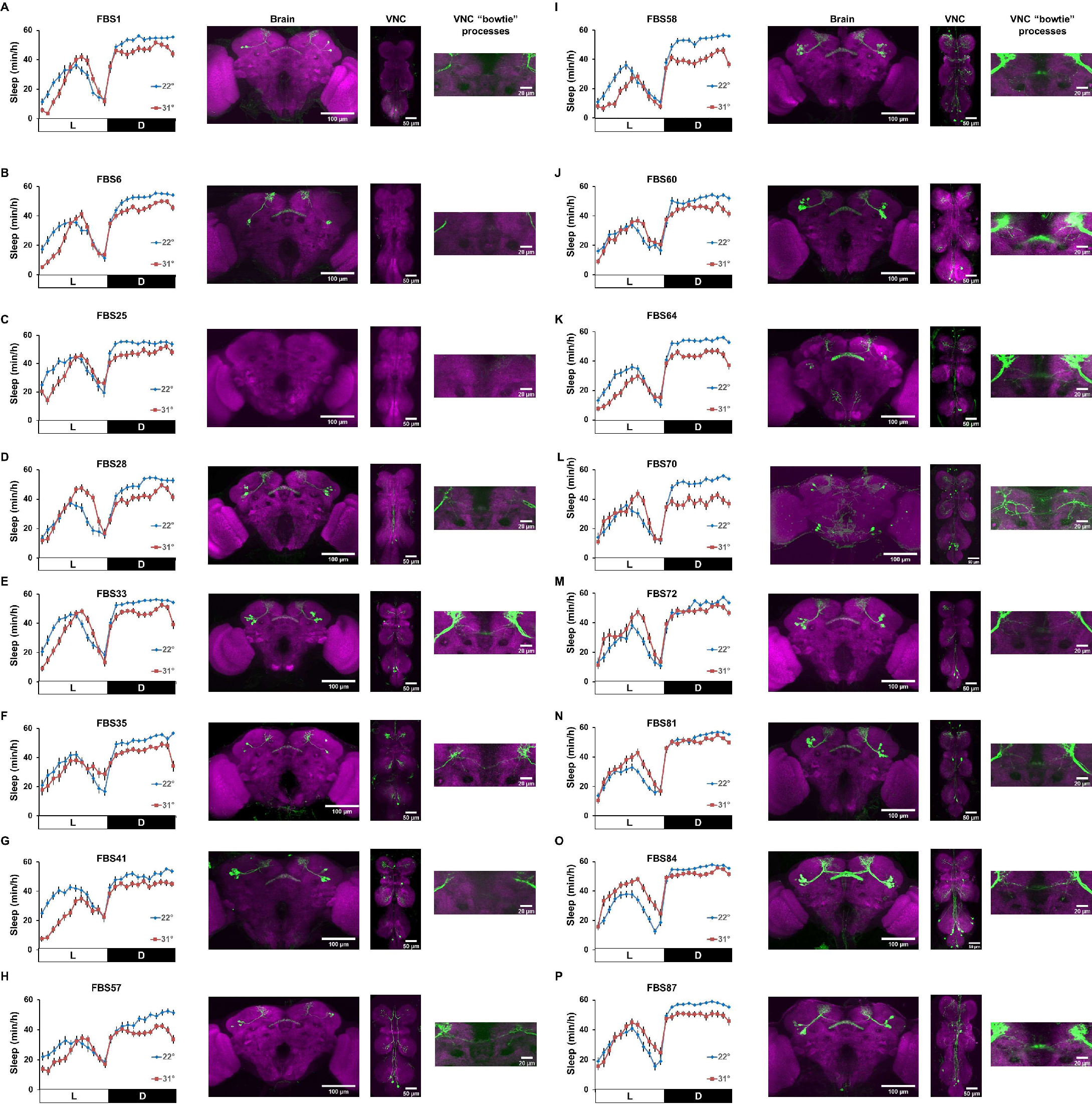

Supplement: S2 Fig — (A–P) Sleep profile (left) and representative confocal stacks (right) for brain and VNC of female FBS>UAS-TrpA1; UAS-mCD8GFP for 16 FBS lines not presented in S1 Fig. (TIF) [file pbio.3003014.s002.tif]

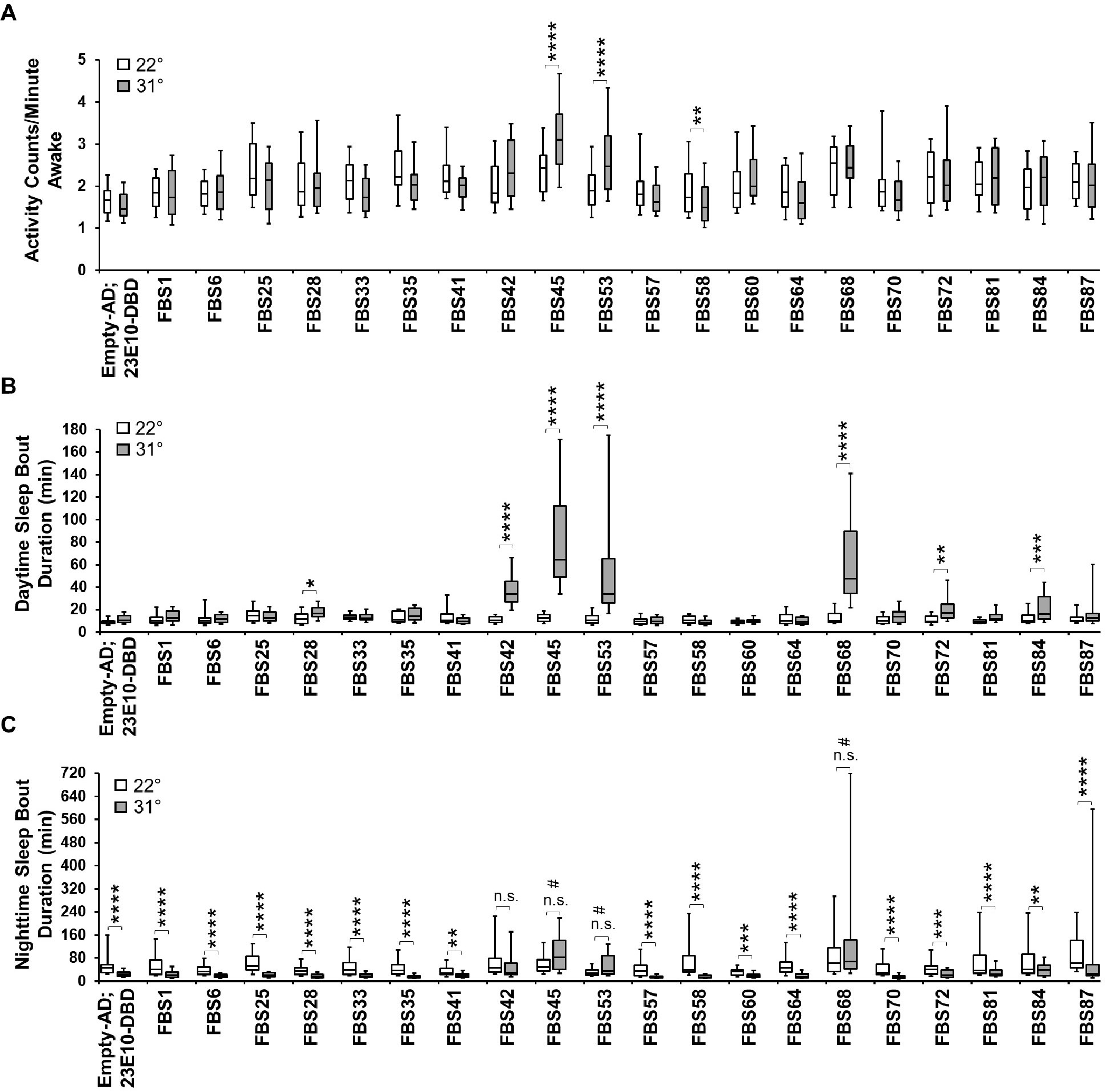

Supplement: S3 Fig — (A) Box plots of locomotor activity counts per minute awake for flies presented in S1B Fig. The bottom and top of each box represents the first and third quartile, and the horizontal line dividing the box is the median. The whiskers represent the 10th and 90th percentiles. Two-way repeated measures ANOVA followed by Sidak’s multiple comparisons test found that for 2 sleep-promoting FBS lines (FBS45 and FBS53) locomotor activity per awake time is increased while no differences are seen for the other 2 sleep-promoting lines between 22 and 31 °C. **P < 0.01, ****P < 0.0001, n = 30–45 flies per genotype. (B) Box plots of daytime sleep bout duration in minutes for flies presented in S1B Fig. Two-way repeated measures ANOVA followed by Sidak’s multiple comparisons test revealed that 7 FBS lines show a significant increase in daytime sleep bout duration between 22 and 31 °C. *P < 0.05, **P < 0.01, ***P < 0.001, ****P < 0.0001, n = 30–45 flies per genotype. (C) Box plots of nighttime sleep bout duration in minutes for flies presented in S1B Fig. Two-way repeated measures ANOVA followed by Sidak’s multiple comparisons test revealed that control and most FBS lines show a significant decrease in nighttime sleep bout duration between 22 and 31 °C. Only 4 sleep-promoting FBS lines show no difference between 22 and 31 °C. Dunnett’s multiple comparisons reveal that for 3 of them, nighttime sleep bout duration at 31 °C is significantly increased compared with Empty control flies (# on figure). **P < 0.01, ***P < 0.001, ****P < 0.0001, n.s. = not significant. n = 30–45 flies per genotype. The raw data underlying parts A, B, and C can be found in S1 Data. (TIF) [file pbio.3003014.s003.tif]

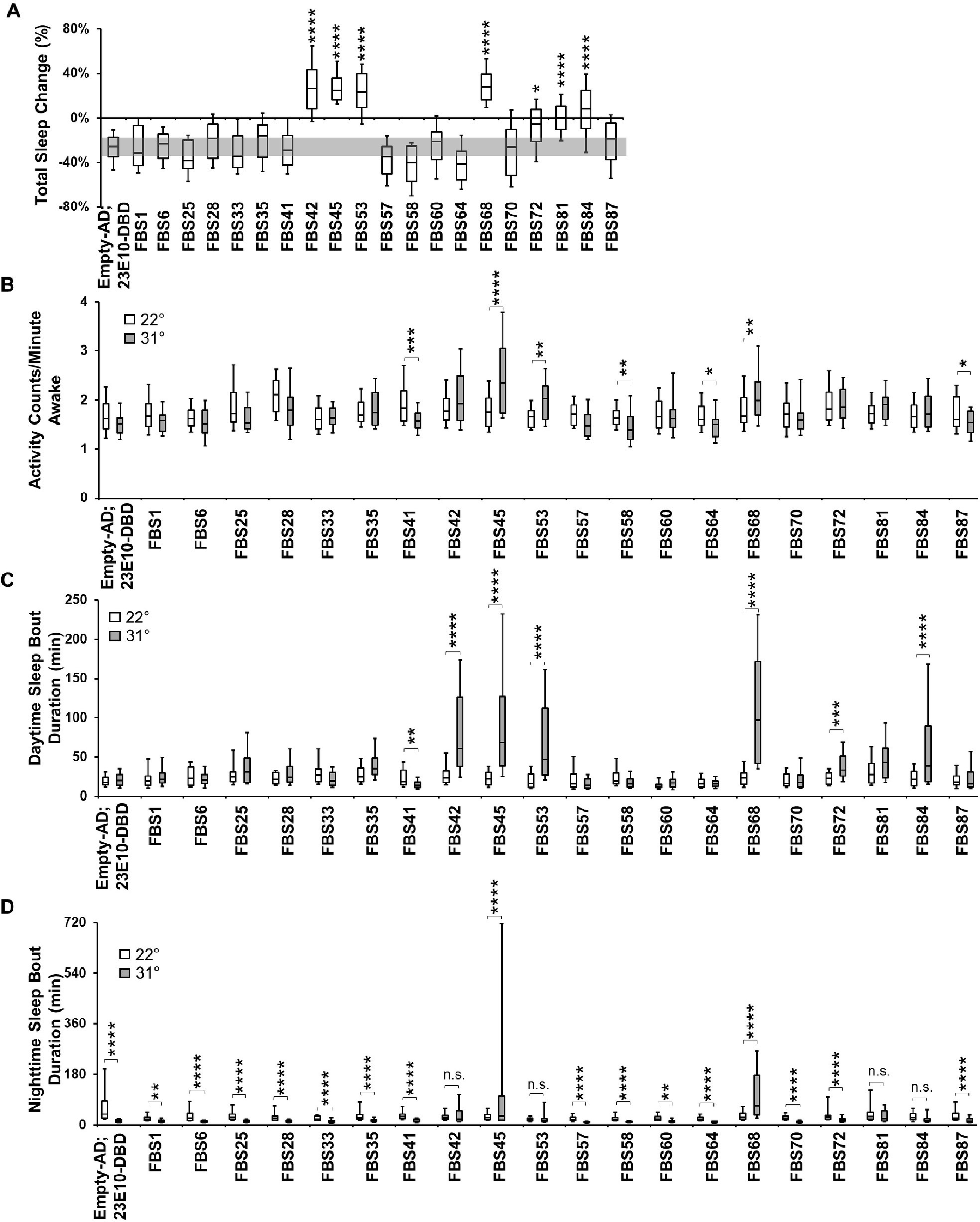

Supplement: S4 Fig — (A) Box plots of total sleep change in % ((total sleep on day 3-total sleep on day 2/total sleep on day 2) × 100) for male control (Empty-AD; 23E10-DBD) and 20 FBS lines expressing UAS-TrpA1; UAS-mCD8GFP. The gray rectangle spanning the horizontal axis indicates the interquartile range of the control. Kruskal–Wallis ANOVA followed by Dunn’s multiple comparisons revealed that 7 FBS lines increase sleep significantly more than control flies when transferred to 31 °C. *P < 0.05, ****P < 0.0001, n = 26–47 flies per genotype. (B) Box plots of locomotor activity counts per minute awake for flies presented in A. Two-way repeated measures ANOVA followed by Sidak’s multiple comparisons test found that for 3 sleep-promoting FBS lines (FBS45, FBS53, and FBS68) locomotor activity per awake time is increased while no differences are seen for the other 4 sleep-promoting lines between 22 and 31 °C. *P < 0.05, **P < 0.01, ***P < 0.001, ****P < 0.0001, n = 26–47 flies per genotype. (C) Box plots of daytime sleep bout duration for flies presented in A. Two-way repeated measures ANOVA followed by Sidak’s multiple comparisons test found that for 6 out of the 7 sleep-promoting FBS lines, daytime sleep bout duration is significantly increased at 31 °C compared with 22 °C. **P < 0.01, ***P < 0.001, ****P < 0.0001, n = 26–47 flies per genotype. (D) Box plots of nighttime sleep bout duration for flies presented in A. Two-way repeated measures ANOVA followed by Sidak’s multiple comparisons revealed that control and most FBS lines show a significant decrease in nighttime sleep bout duration between 22 °C and 31 °C, and 4 sleep-promoting FBS lines show no difference between 22 and 31 °C (FBS42, FBS53, FBS81, and FBS84) while FBS45 and FBS68 show an increase in nighttime sleep bout duration at 31 °C. **P < 0.01, ****P < 0.0001, n = 26–47 flies per genotype. The raw data underlying parts A, B, C, and D can be found in S1 Data. (TIF) [file pbio.3003014.s004.tif]

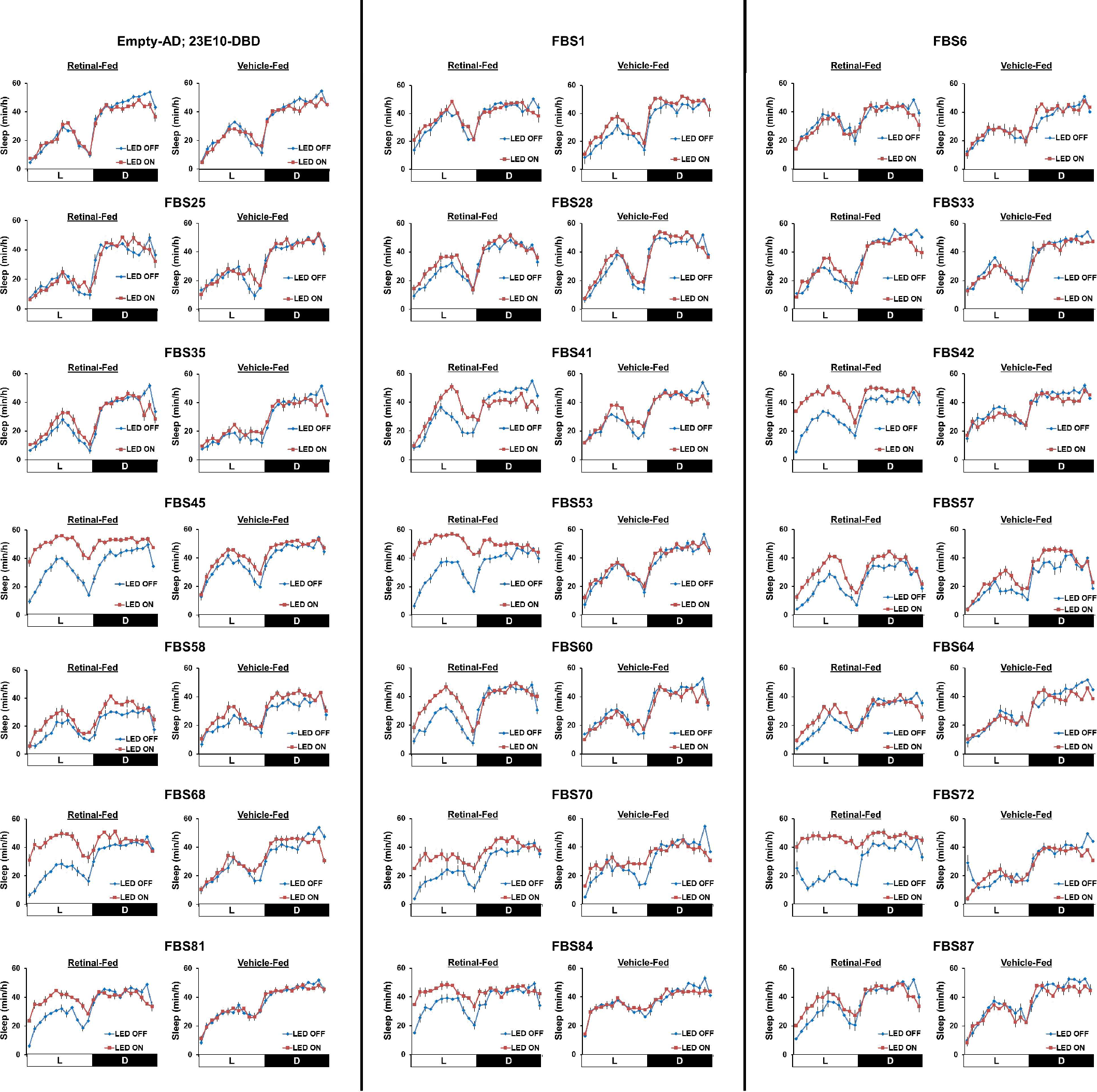

Supplement: S5 Fig — (TIF) [file pbio.3003014.s005.tif]

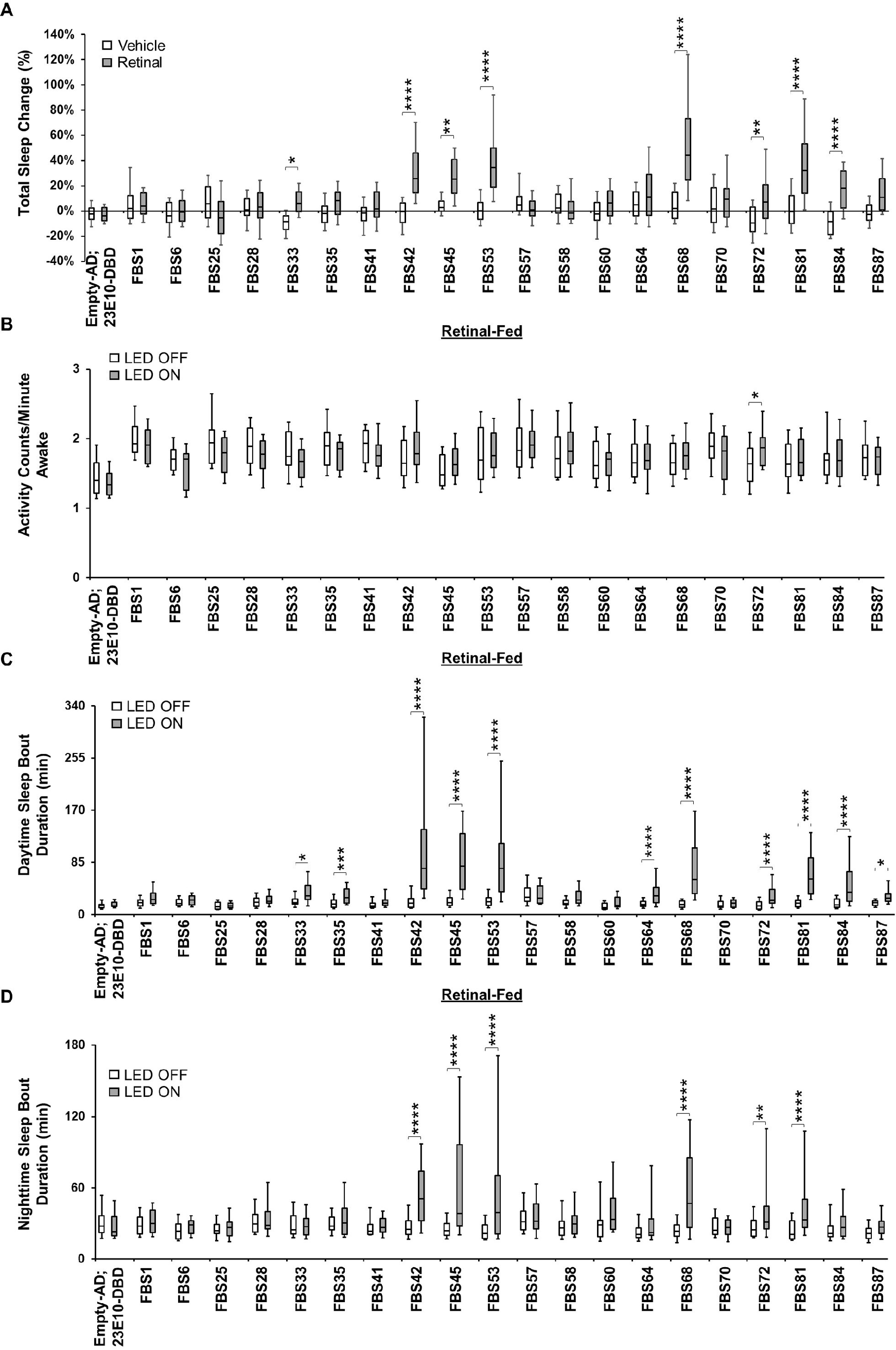

Supplement: S6 Fig — . (A) Box plots of total sleep change in % ((total sleep on day 3-total sleep on day 2/total sleep on day 2) × 100) for control (Empty) and 20 experimental vehicle-fed and retinal-fed male flies expressing CsChrimson upon 627 nm LED stimulation. Two-way ANOVA followed by Sidak’s multiple comparisons revealed that 8 retinal-fed FBS lines increase sleep significantly when stimulated with 627 nm LEDs when compared with vehicle-fed flies. *P < 0.05, **P < 0.01, ****P < 0.0001. n = 20–44 flies per genotype and condition. (B) Box plots of locomotor activity counts per minute awake for retinal-fed flies presented in A. Two-way repeated measures ANOVA followed by Sidak’s multiple comparisons test found that for most sleep-promoting FBS lines, locomotor activity per awake time is not affected when the flies are stimulated with 627 nm LEDs while it is increased in FBS72>UAS-CsChrimson flies. *P < 0.05, n = 24–44 flies per genotype. (C) Box plots of daytime sleep bout duration (in minutes) for retinal-fed flies presented in A. Two-way repeated measures ANOVA followed by Sidak’s multiple comparisons indicate that daytime sleep bout duration is increased in 11 FBS lines expressing CsChrimson when stimulated with 627 nm LEDs. *P < 0.05, ***P < 0.001, ****P < 0.0001, n = 24–44 flies per genotype. (D) Box plots of nighttime sleep bout duration (in minutes) for retinal-fed flies presented in A. Two-way repeated measures ANOVA followed by Sidak’s multiple comparisons indicate that nighttime sleep bout duration is increased in 6 FBS lines expressing CsChrimson when stimulated with 627 nm LEDs. **P < 0.01, ****P < 0.0001, n = 24–44 flies per genotype. The raw data underlying parts A, B, C, and D can be found in S1 Data. (TIF) [file pbio.3003014.s006.tif]

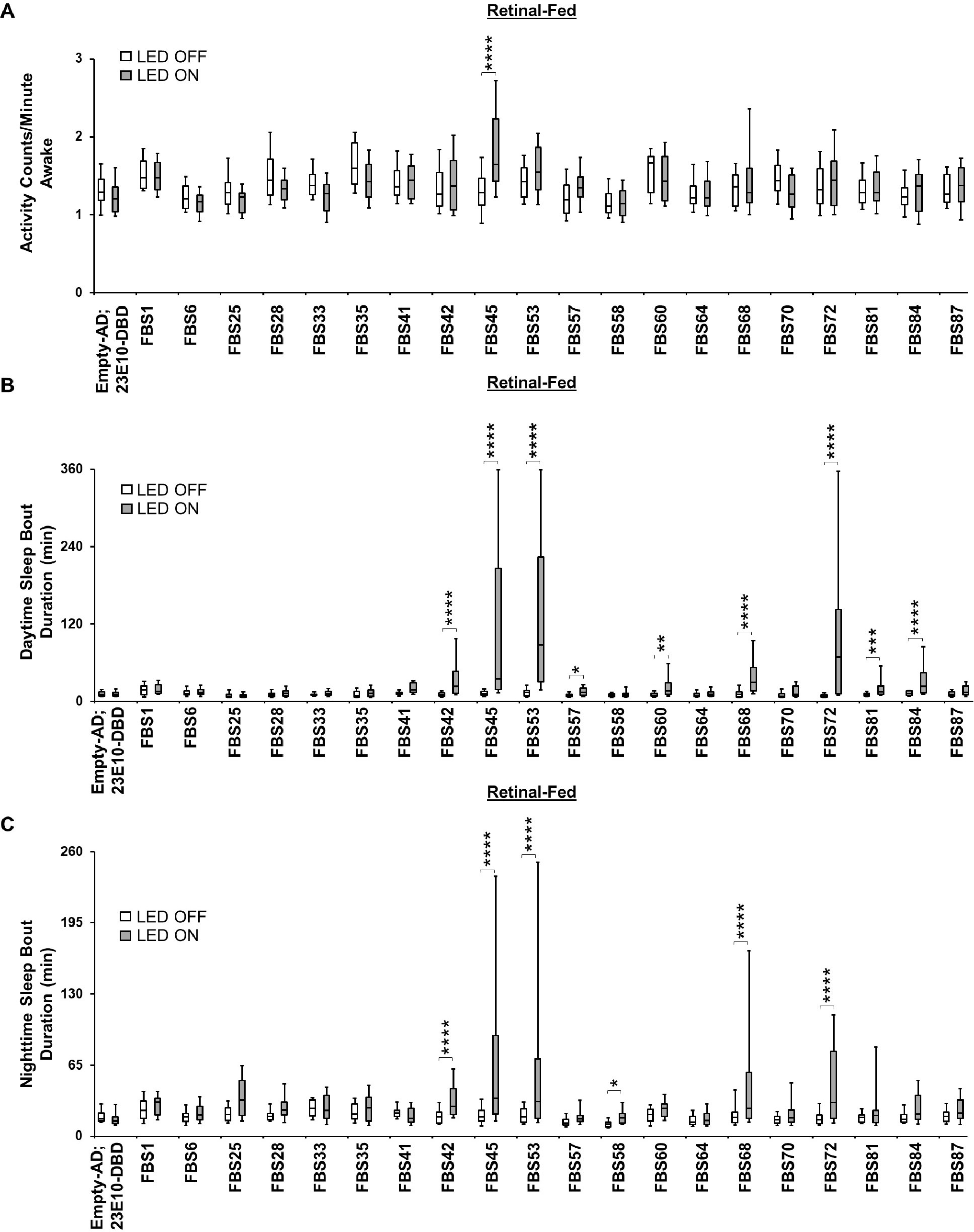

Supplement: S7 Fig — (A) Box plots of locomotor activity counts per minute awake for retinal-fed flies presented in S1C Fig. The bottom and top of each box represents the first and third quartile, and the horizontal line dividing the box is the median. The whiskers represent the 10th and 90th percentiles. Two-way repeated measures ANOVA followed by Sidak’s multiple comparisons test found that for most sleep-promoting FBS lines, locomotor activity per awake time is not affected when the flies are stimulated with 627 nm LEDs while it is increased in FBS45>UAS-CsChrimson flies. ****P < 0.0001, n = 21–40 flies per genotype. (B) Box plots of daytime sleep bout duration (in minutes) for retinal-fed flies presented in S1C Fig. The bottom and top of each box represents the first and third quartile, and the horizontal line dividing the box is the median. The whiskers represent the 10th and 90th percentiles. Two-way repeated measures ANOVA followed by Sidak’s multiple comparisons indicate that daytime sleep bout duration is increased in 9 FBS lines expressing CsChrimson when stimulated with 627 nm LEDs. *P < 0.05, **P < 0.01, ***P < 0.001, ****P < 0.0001, n = 21–40 flies per genotype. (C) Box plots of nighttime sleep bout duration (in minutes) for retinal-fed flies presented in S1C Fig. The bottom and top of each box represents the first and third quartile, and the horizontal line dividing the box is the median. The whiskers represent the 10th and 90th percentiles. Two-way repeated measures ANOVA followed by Sidak’s multiple comparisons indicate that nighttime sleep bout duration is increased in 6 FBS lines expressing CsChrimson when stimulated with 627 nm LEDs. *P < 0.05, ****P < 0.0001, n = 21–40 flies per genotype. The raw data underlying parts A, B, and C can be found in S1 Data. (TIF) [file pbio.3003014.s007.tif]

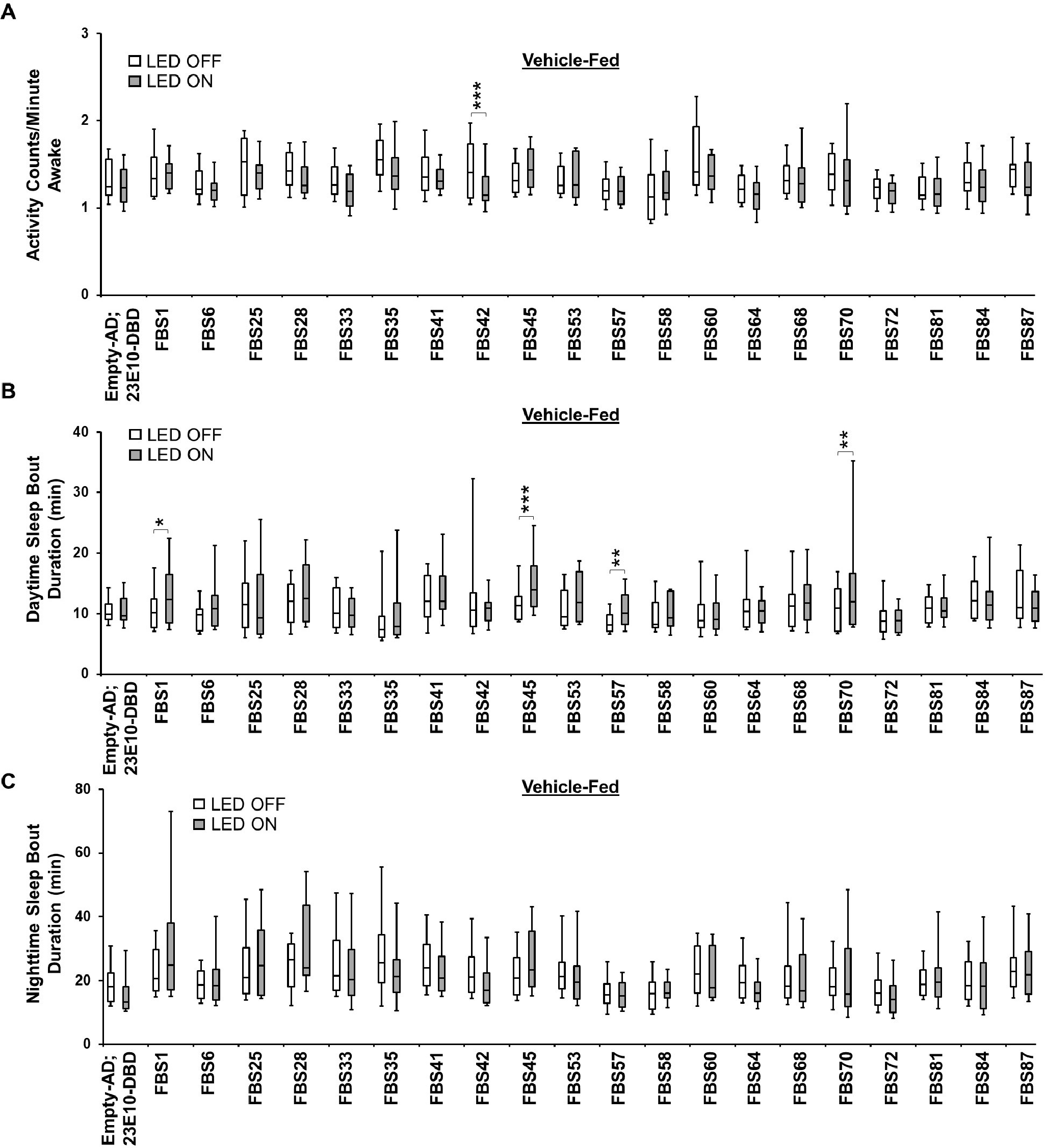

Supplement: S8 Fig — (A) Box plots of locomotor activity counts per minute awake for vehicle-fed flies presented in S1C Fig. Two-way repeated measures ANOVA followed by Sidak’s multiple comparisons test found that all lines except FBS42 show no difference in locomotor activity per awake time when the flies are stimulated with 627 nm LEDs. ***P < 0.001. n = 20–39 flies per genotype. (B) Box plots of daytime sleep bout duration for vehicle-fed flies presented in S1C Fig. Two-way repeated measures ANOVA followed by Sidak’s multiple comparisons test found that most vehicle-fed sleep-promoting lines show no difference in daytime sleep bout duration when the flies are stimulated with 627 nm LEDs. *P < 0.05, **P < 0.01, ***P < 0.001. n = 20–39 flies per genotype. (C) Box plots of nighttime sleep bout duration for vehicle-fed flies presented in S1C Fig. Two-way repeated measures ANOVA followed by Sidak’s multiple comparisons test show no difference in nighttime sleep bout duration when vehicle-fed flies are stimulated with 627 nm LEDs. n = 20–39 flies per genotype. The raw data underlying parts A, B, and C can be found in S1 Data. (TIF) [file pbio.3003014.s008.tif]

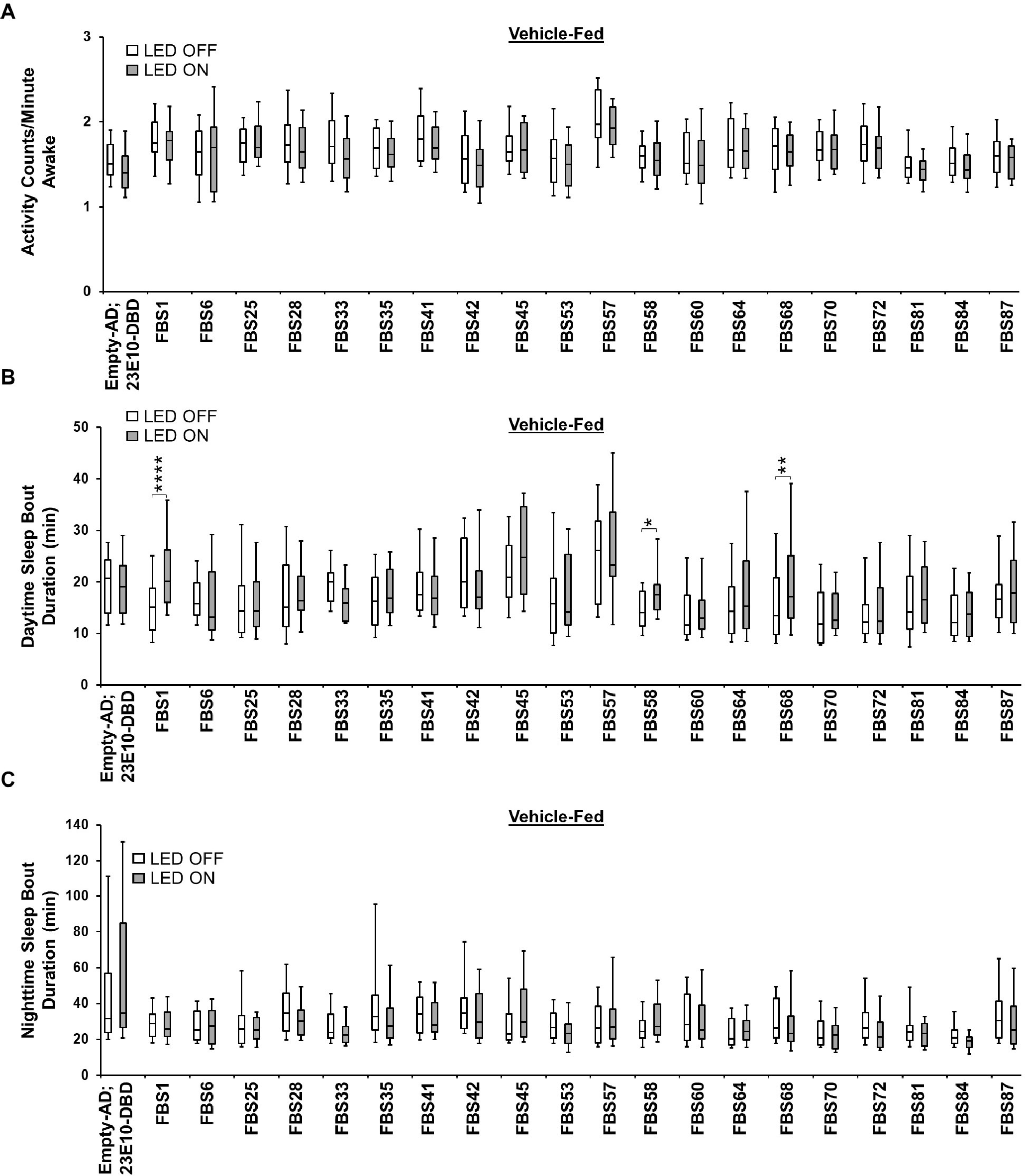

Supplement: S9 Fig — (A) Box plots of locomotor activity counts per minute awake for vehicle-fed flies presented in S6A Fig. Two-way repeated measures ANOVA followed by Sidak’s multiple comparisons test found no difference in locomotor activity per awake time when the flies are stimulated with 627 nm LEDs. n = 20–40 flies per genotype. (B) Box plots of daytime sleep bout duration for vehicle-fed flies presented in S6A Fig. Two-way repeated measures ANOVA followed by Sidak’s multiple comparisons test found that most vehicle-fed sleep-promoting lines show no difference in daytime sleep bout duration when the flies are stimulated with 627 nm LEDs. *P < 0.05, **P < 0.01, ****P < 0.0001. n = 20–40 flies per genotype. (C) Box plots of nighttime sleep bout duration for vehicle-fed flies presented in S6A Fig. Two-way repeated measures ANOVA followed by Sidak’s multiple comparisons test show no difference in nighttime sleep bout duration when vehicle-fed flies are stimulated with 627 nm LEDs. n = 20–40 flies per genotype. The raw data underlying parts A, B, and C can be found in S1 Data. (TIF) [file pbio.3003014.s009.tif]

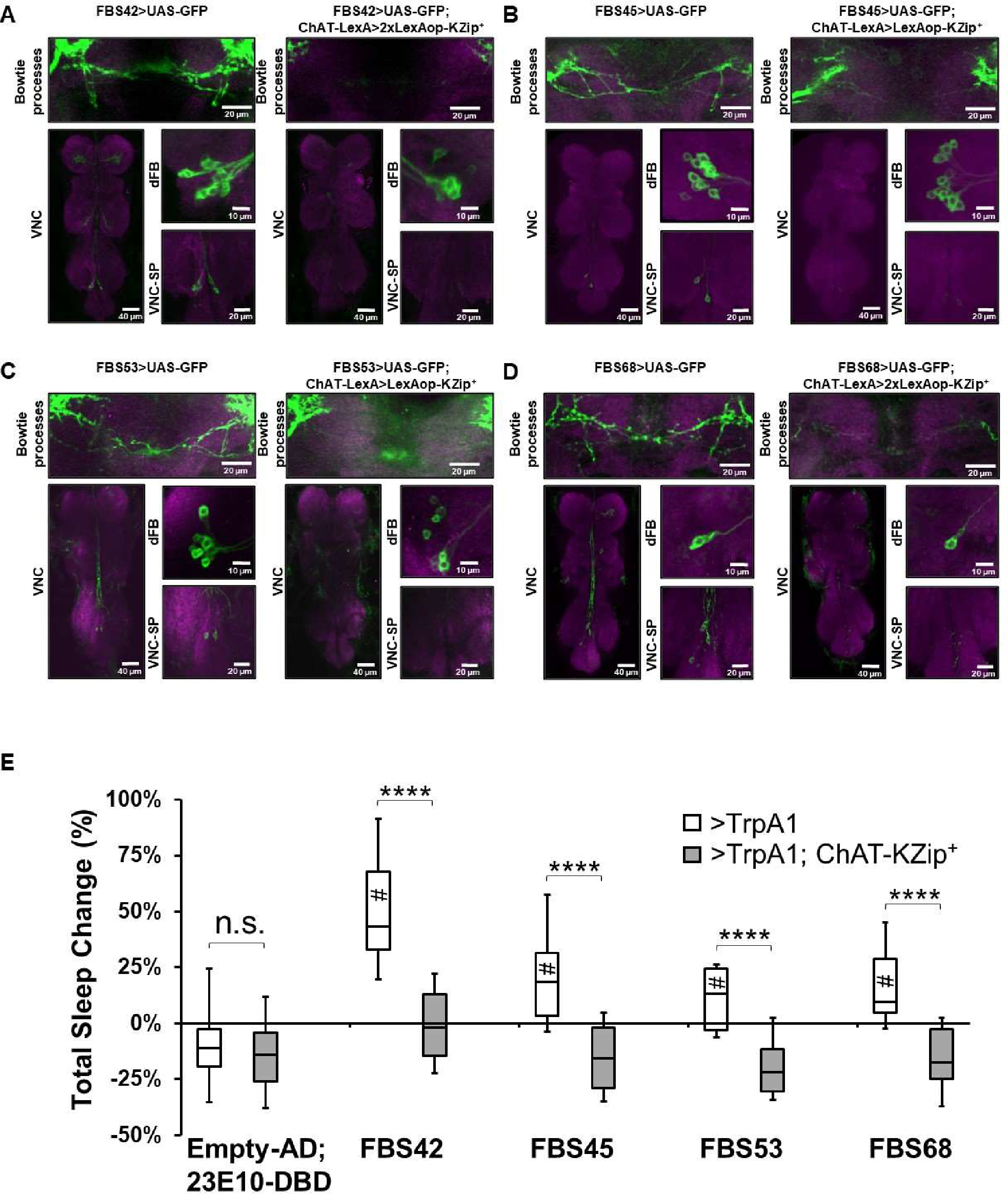

Supplement: S10 Fig — (A) Representative confocal stacks of FBS42>UAS-GFP (left) and FBS42>UAS-GFP; ChAT-LexA>2x LexAop2KZip+ (right) female flies showing the location of the VNC-SP bowtie processes, the dFB region, the VNC and VNC-SP neurons. Expression of GFP in VNC-SP neurons and in their bowtie brain processes is abolished by the expression of the KZip+ repressor. Gray arrows show VNC-SP neurons. Green, anti-GFP; magenta, anti-nc82. (B) Representative confocal stacks of FBS45>UAS-GFP (left) and FBS45>UAS-GFP; ChAT-LexA> LexAop2KZip+ (right) female flies showing the location of the VNC-SP bowtie processes, the dFB region, the VNC and VNC-SP neurons. Expression of GFP in VNC-SP neurons and in their bowtie brain processes is abolished by the expression of the KZip+ repressor. Gray arrows show VNC-SP neurons. Green, anti-GFP; magenta, anti-nc82. (C) Representative confocal stacks of FBS53>UAS-GFP (left) and FBS53>UAS-GFP; ChAT-LexA> LexAop2KZip+ (right) female flies showing the location of the VNC-SP bowtie processes, the dFB region, the VNC and VNC-SP neurons. Expression of GFP in VNC-SP neurons and in their bowtie brain processes is abolished by the expression of the KZip+ repressor. Gray arrows show VNC-SP neurons. Green, anti-GFP; magenta, anti-nc82. (D) Representative confocal stacks of FBS68>UAS-GFP (left) and FBS68>UAS-GFP; ChAT-LexA> 2x LexAop2KZip+ (right) female flies showing the location of the VNC-SP bowtie processes, the dFB region, the VNC and VNC-SP neurons. Expression of GFP in VNC-SP neurons and in their bowtie brain processes is abolished by the expression of the KZip+ repressor. Gray arrows show VNC-SP neurons. Green, anti-GFP; magenta, anti-nc82. (E) Box plots of total sleep change in % ((total sleep on day 3-total sleep on day 2/total sleep on day 2) × 100) for female control (Empty: Empty-AD; 23E10-DBD) and 4 sleep-promoting FBS lines expressing UAS-TrpA1 or UAS-TrpA1 and the KZip+ repressor in cholinergic neurons (ChAT-KZip+). Two-way ANOVA followed by Sidak’s mult [file pbio.3003014.s010.tif]

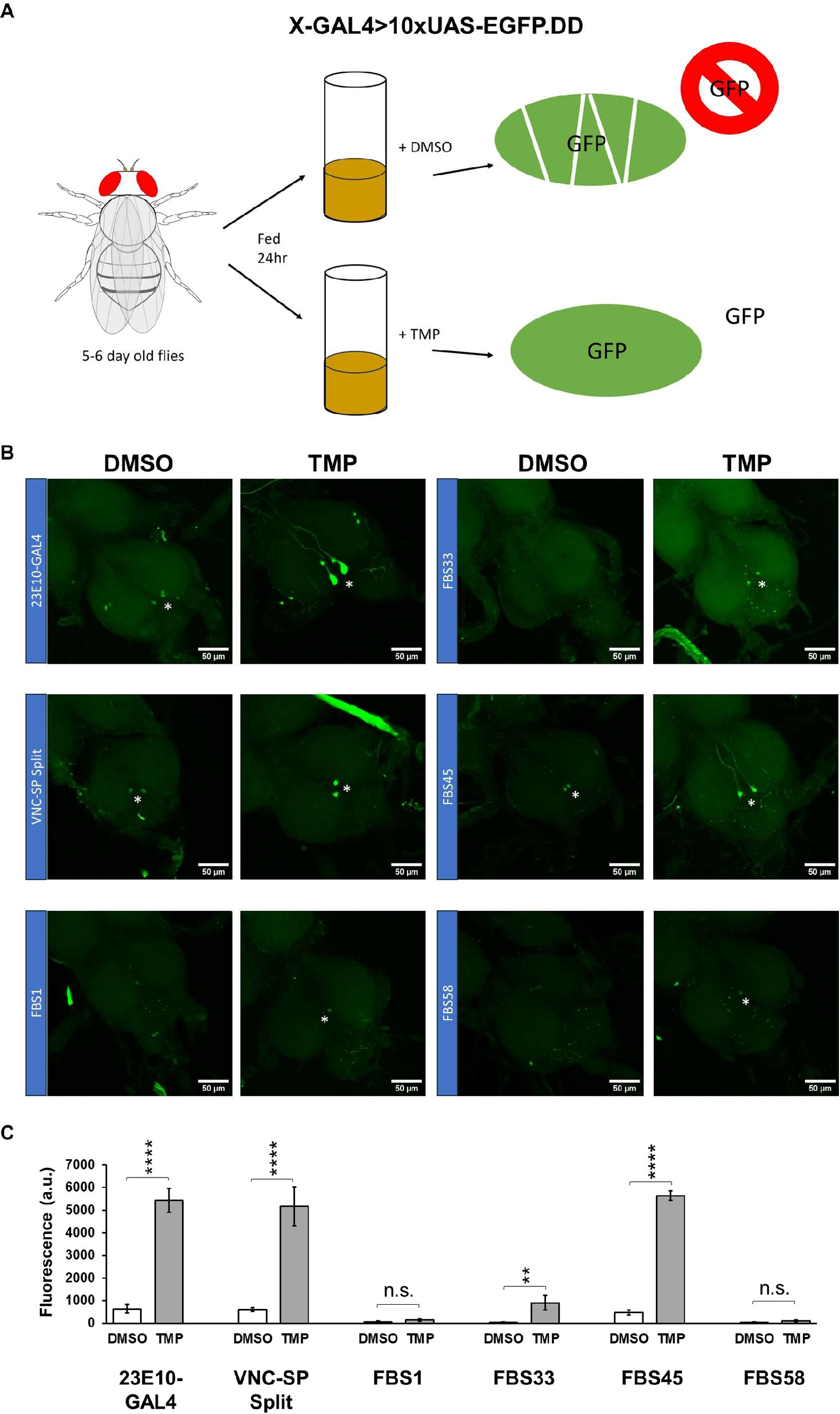

Supplement: S11 Fig — (A) Schematic of the GFP.DD experimental design. (B) Representative confocal stacks of females 23E10-GAL4, VNC-SP Split, FBS1, FBS33, FBS45, and FBS58 expressing GFP-DD and fed DMSO or TMP for 24 h before dissection. Stacks are focused on the metathoracic region of the VNC where VNC-SP cell bodies are located. Green, anti-GFP. White asterisks indicate VNC-SP cell bodies. (C) Quantification of staining intensity for data presented in B. Two-way ANOVA followed by Sidak’s multiple comparisons revealed that there is significantly more GFP staining in VNC-SP neurons in 23E10-GAL4, VNC-SP Split, FBS33, and FBS45 fed TMP compared with DMSO fed flies. n.s = not significant, **P < 0.01, ****P < 0.0001. n = 5–14 VNC per genotype and condition. The raw data underlying part C can be found in S1 Data. (TIF) [file pbio.3003014.s011.tif]

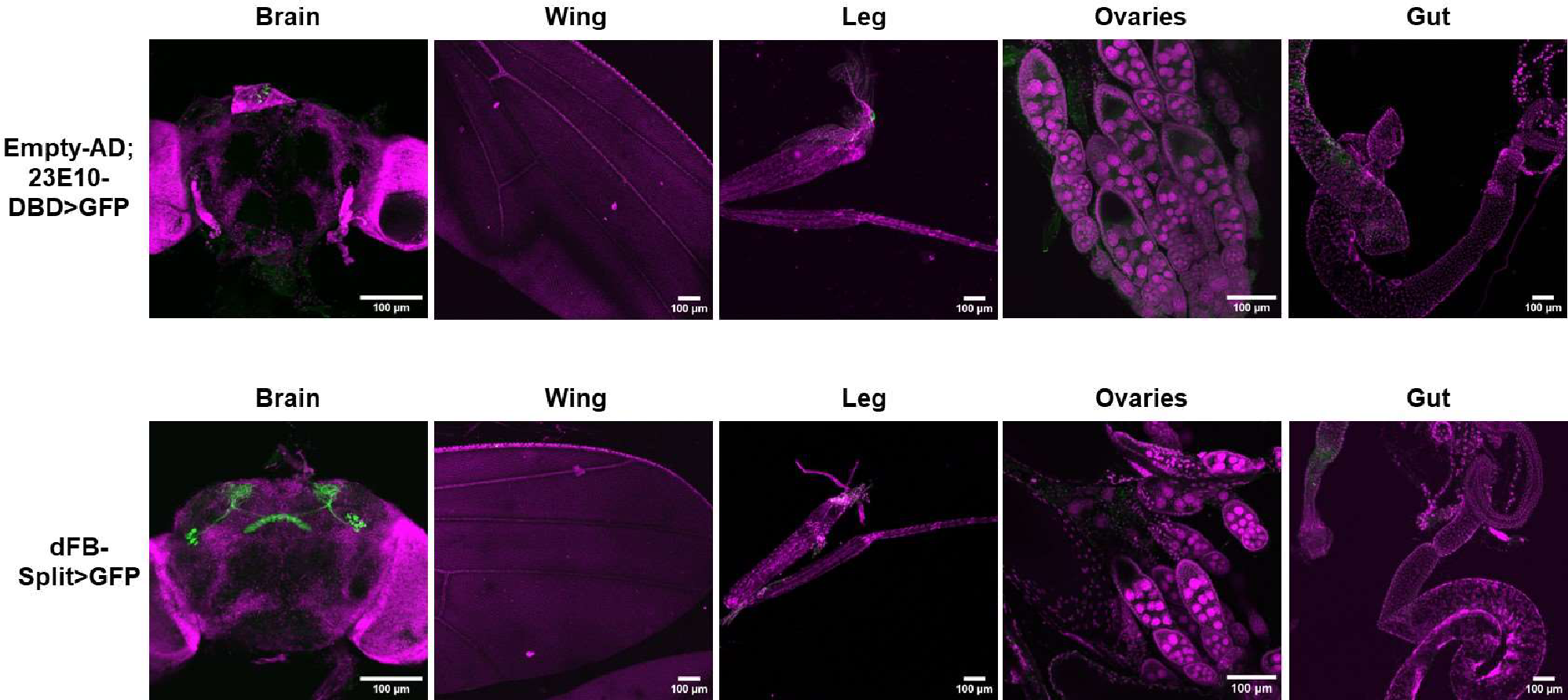

Supplement: S12 Fig — GFP is expressed in the brain, but not the wings, legs, ovaries, or gut in dFB-Split. Tissue was dissected, fixed, and stained with DAPI. Green, anti-GFP; Blue, DAPI. (TIF) [file pbio.3003014.s012.tif]

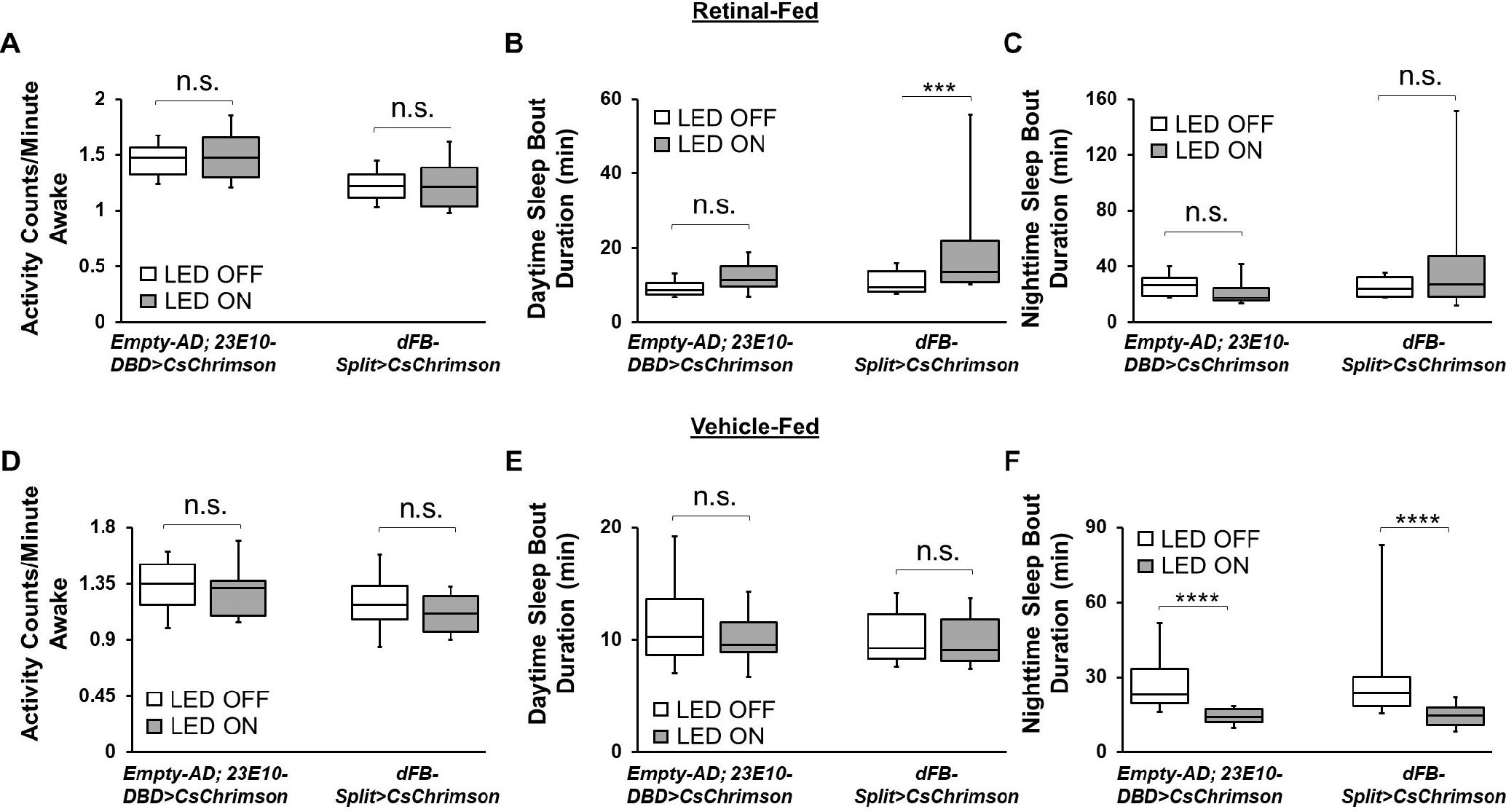

Supplement: S13 Fig — (A) Box plots of locomotor activity counts per minute awake for retinal-fed flies presented in Fig 1G. The bottom and top of each box represents the first and third quartile, and the horizontal line dividing the box is the median. The whiskers represent the 10th and 90th percentiles. Two-way repeated measures ANOVA followed by Sidak’s multiple comparisons test found no difference in locomotor activity per awake time when the flies are stimulated at 10 Hz with 627 nm LEDs. n.s = not significant. n = 23–24 flies per genotype. (B) Box plots of daytime sleep bout duration (in minutes) for retinal-fed flies presented in Fig 1G. The bottom and top of each box represents the first and third quartile, and the horizontal line dividing the box is the median. The whiskers represent the 10th and 90th percentiles. Two-way repeated measures ANOVA followed by Sidak’s multiple comparisons indicate that daytime sleep bout duration is increased in dFB-Split>CsChrimson female flies when stimulated with 627 nm LEDs at 10 Hz. n.s = not significant, ***P < 0.001. n = 23–24 flies per genotype. (C) Box plots of nighttime sleep bout duration (in minutes) for retinal-fed flies presented in Fig 1G. The bottom and top of each box represents the first and third quartile, and the horizontal line dividing the box is the median. The whiskers represent the 10th and 90th percentiles. Two-way repeated measures ANOVA followed by Sidak’s multiple comparisons found no differences in nighttime sleep bout duration. n.s = not significant. n = 23–24 flies per genotype. (D) Box plots of locomotor activity counts per minute awake for vehicle-fed flies presented in Fig 1G. The bottom and top of each box represents the first and third quartile, and the horizontal line dividing the box is the median. The whiskers represent the 10th and 90th percentiles. Two-way repeated measures ANOVA followed by Sidak’s multiple comparisons test found no difference in locomotor activity per awake time when the flies are stimula [file pbio.3003014.s013.tif]

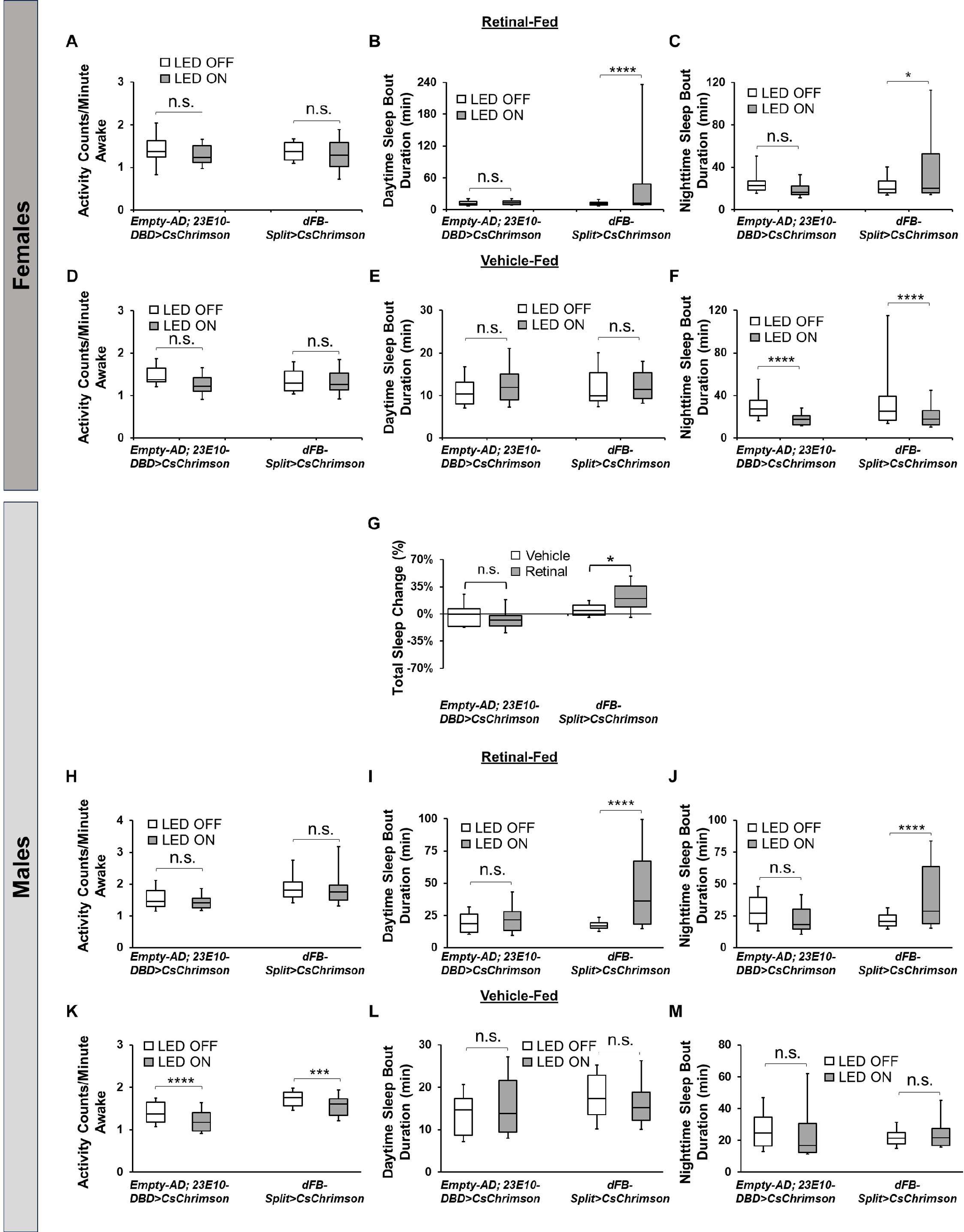

Supplement: S14 Fig — (A) Box plots of locomotor activity counts per minute awake for retinal-fed flies presented in Fig 1I. Two-way repeated measures ANOVA followed by Sidak’s multiple comparisons test found no difference in locomotor activity per awake time when the flies are stimulated with 627 nm LEDs. n.s. = not significant. n = 34–38 flies per genotype. (B) Box plots of daytime sleep bout duration for retinal-fed flies presented in Fig 1I. Two-way repeated measures ANOVA followed by Sidak’s multiple comparisons test found that daytime sleep bout duration is significantly increased in dFB-Split>CsChrimson female flies that are stimulated with 627 nm LEDs. ****P < 0.0001, n.s. = not significant. n = 34–38 flies per genotype. (C) Box plots of nighttime sleep bout duration for retinal-fed flies presented in Fig 1I. Two-way repeated measures ANOVA followed by Sidak’s multiple comparisons test found that nighttime sleep bout duration is significantly increased in dFB-Split>CsChrimson female flies that are stimulated with 627 nm LEDs. *P < 0.05, n.s. = not significant. n = 34–38 flies per genotype. (D) Box plots of locomotor activity counts per minute awake for vehicle-fed flies presented in Fig 1I. Two-way repeated measures ANOVA followed by Sidak’s multiple comparisons test found no difference in locomotor activity per awake time when the flies are stimulated with 627 nm LEDs. n.s. = not significant. n = 26–32 flies per genotype. (E) Box plots of daytime sleep bout duration for vehicle-fed flies presented in Fig 1I. Two-way repeated measures ANOVA followed by Sidak’s multiple comparisons test found no difference in daytime sleep bout duration when the flies are stimulated with 627 nm LEDs. n.s. = not significant. n = 26–32 flies per genotype. (F) Box plots of nighttime sleep bout duration for vehicle-fed flies presented in Fig 1I. Two-way repeated measures ANOVA followed by Sidak’s multiple comparisons test. ****P < 0.0001. n = 26–32 flies per genotype. (G) Box plots of total sleep chan [file pbio.3003014.s014.tif]

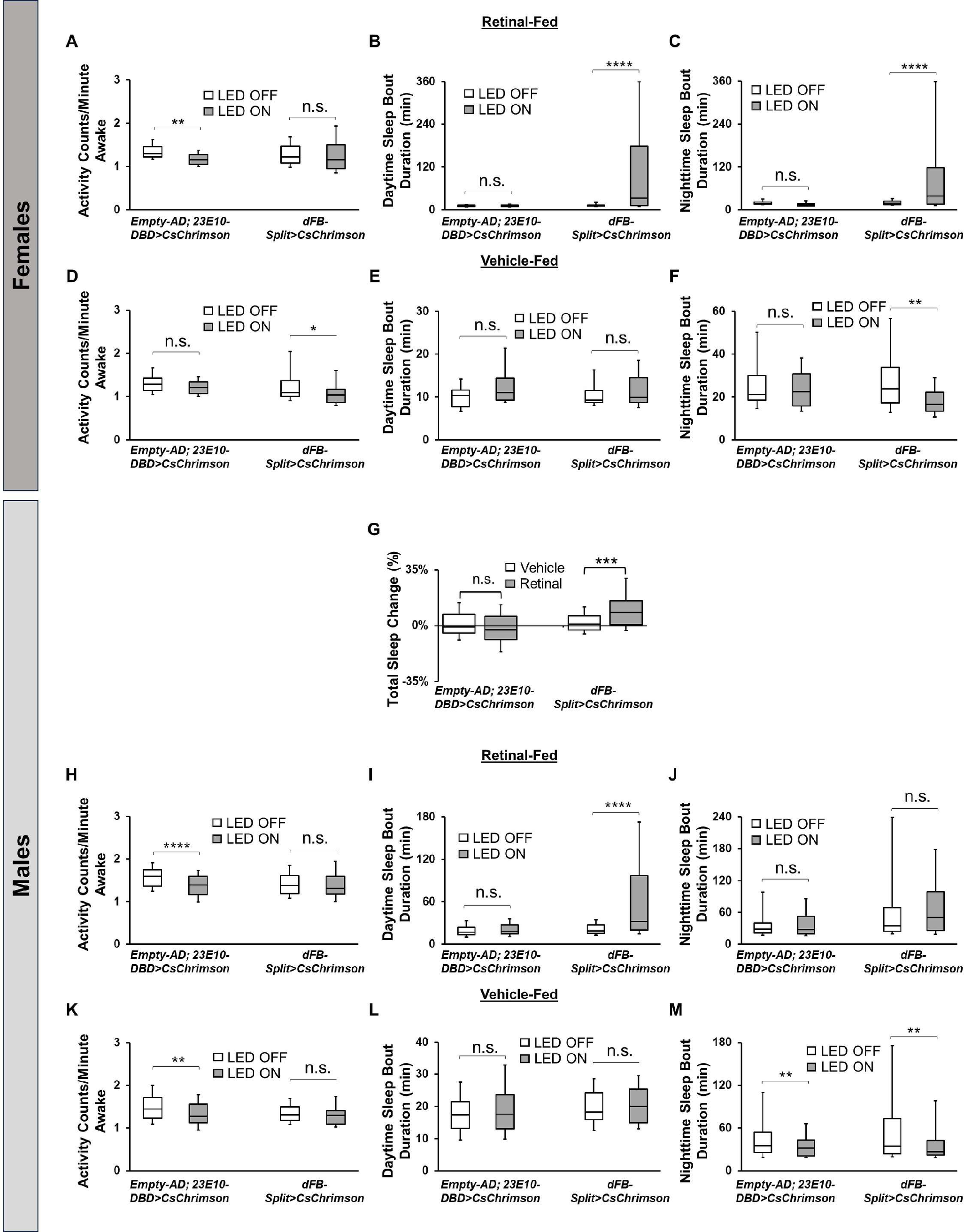

Supplement: S15 Fig — (A) Box plots of locomotor activity counts per minute awake for retinal-fed flies presented in Fig 1K. Two-way repeated measures ANOVA followed by Sidak’s multiple comparisons test found no difference in locomotor activity per awake time in dFB-Split>CsChrimson female flies that are stimulated with 627 nm LEDs. **P < 0.01, n.s. = not significant. n = 33–38 flies per genotype. (B) Box plots of daytime sleep bout duration for retinal-fed flies presented in Fig 1K. Two-way repeated measures ANOVA followed by Sidak’s multiple comparisons test found that daytime sleep bout duration is significantly increased in dFB-Split>CsChrimson female flies that are stimulated with 627 nm LEDs. ****P < 0.0001, n.s. = not significant. n = 33–38 flies per genotype. (C) Box plots of nighttime sleep bout duration for retinal-fed flies presented in Fig 1K. Two-way repeated measures ANOVA followed by Sidak’s multiple comparisons test found that nighttime sleep bout duration is significantly increased in dFB-Split>CsChrimson female flies that are stimulated with 627 nm LEDs. ****P < 0.0001, n.s. = not significant. n = 33–38 flies per genotype. (D) Box plots of locomotor activity counts per minute awake for vehicle-fed flies presented in Fig 1K. Two-way repeated measures ANOVA followed by Sidak’s multiple comparisons test. *P < 0.05, n.s. = not significant. n = 25–31 flies per genotype. (E) Box plots of daytime sleep bout duration for vehicle-fed flies presented in Fig 1K. Two-way repeated measures ANOVA followed by Sidak’s multiple comparisons test found no difference in daytime sleep bout duration when the flies are stimulated with 627 nm LEDs. n.s. = not significant. n = 25–31 flies per genotype. (F) Box plots of nighttime sleep bout duration for vehicle-fed flies presented in Fig 1K. Two-way repeated measures ANOVA followed by Sidak’s multiple comparisons test. **P < 0.01, n.s. = not significant. n = 25–31 flies per genotype. (G) Box plots of total sleep change in % for male control (Emp [file pbio.3003014.s015.tif]

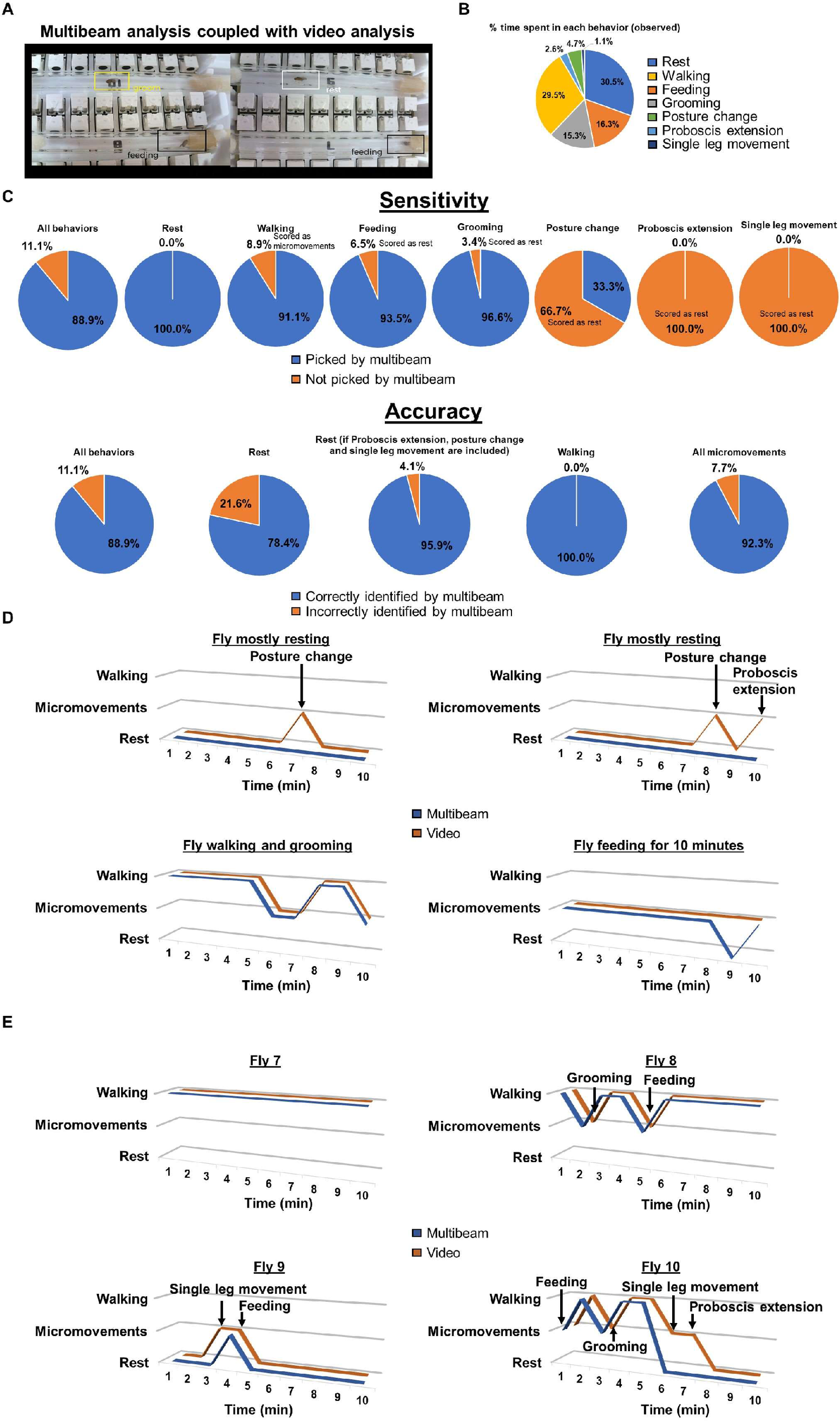

Supplement: S16 Fig — (A) Setup for combined multibeam and video analysis. (B) Observed % time spent in each of 7 behaviors (Rest, Walking, Feeding, Grooming, Posture change, Proboscis extension, and single leg movement) for 19 Canton-S flies during 10 min recording. (C) Sensitivity (ratio of minutes with a behavior identified by the multibeam system to all visually labeled minutes of the same behavior) and accuracy (ratio of correctly identified minutes with a behavior by the multibeam system to all identified minutes with the same behavior by the multibeam system) of the multibeam system for identifying all behaviors. For the accuracy calculation, all micromovements (Feeding, Grooming, Posture change, Proboscis extension, and single leg movement) were pooled together. (D) Comparison of multibeam analysis and video observation for 4 Canton-S flies during 10 min. (E) Comparison of multibeam analysis and video observation for the 4 Canton-S flies shown in Movie 1. The raw data underlying parts B, C, and E can be found in S1 Data. (TIF) [file pbio.3003014.s016.tif]

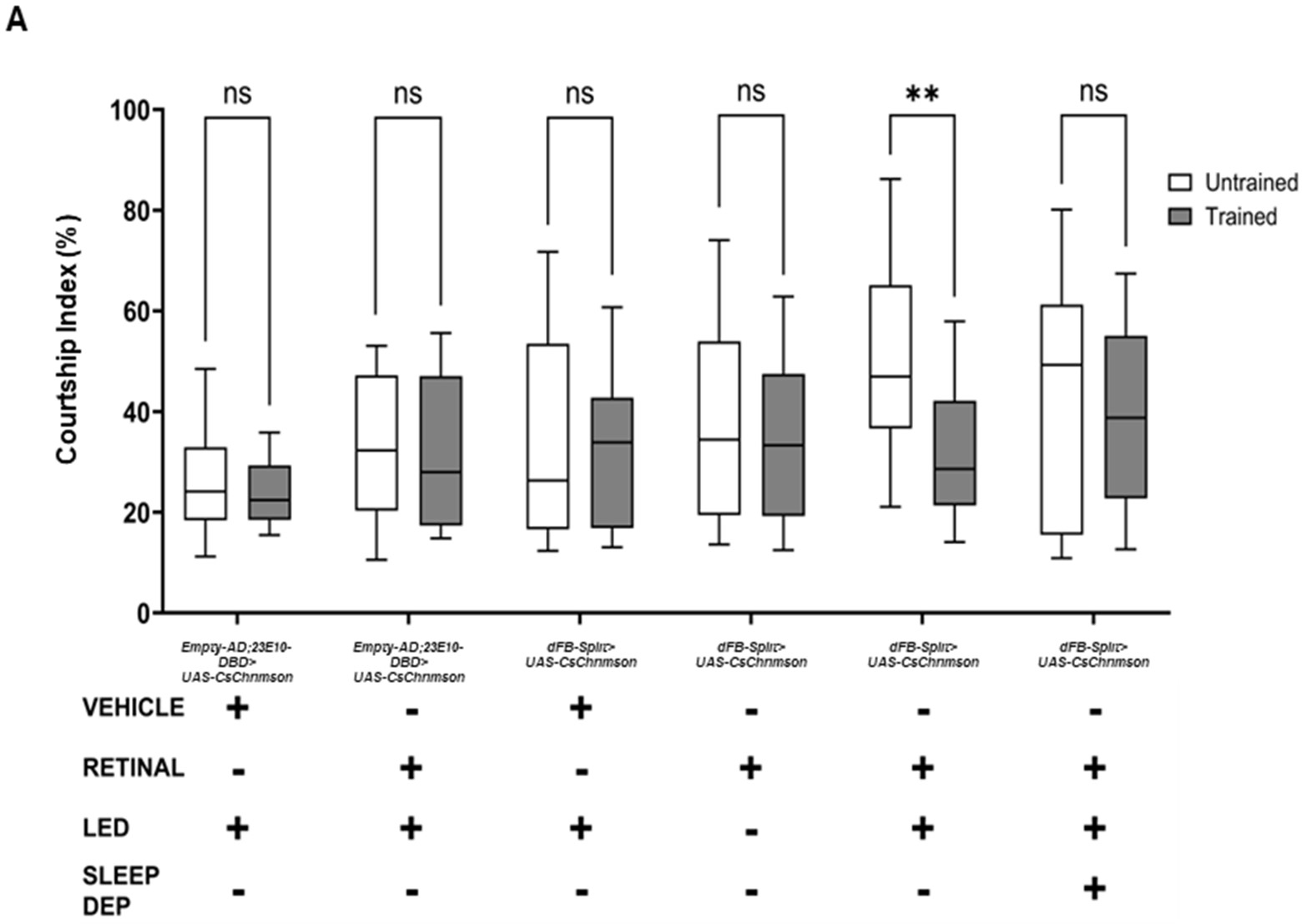

Supplement: S17 Fig — (A) Courtship index values for male flies in untrained and trained groups for each condition presented in Fig 2C. Unpaired parametric t test for each group of untrained and trained males for each condition. Sample size (untrained:trained) from left to right on the graph, n = 54 (27:27), 51 (27:24), 49 (27:22), 51 (26:25), 46 (21:25), and 53 (27:26), respectively. Courtship indices <10% were excluded from both groups. **P < 0.01, ns = not significant. The raw data underlying part A can be found in S1 Data. (TIF) [file pbio.3003014.s017.tif]

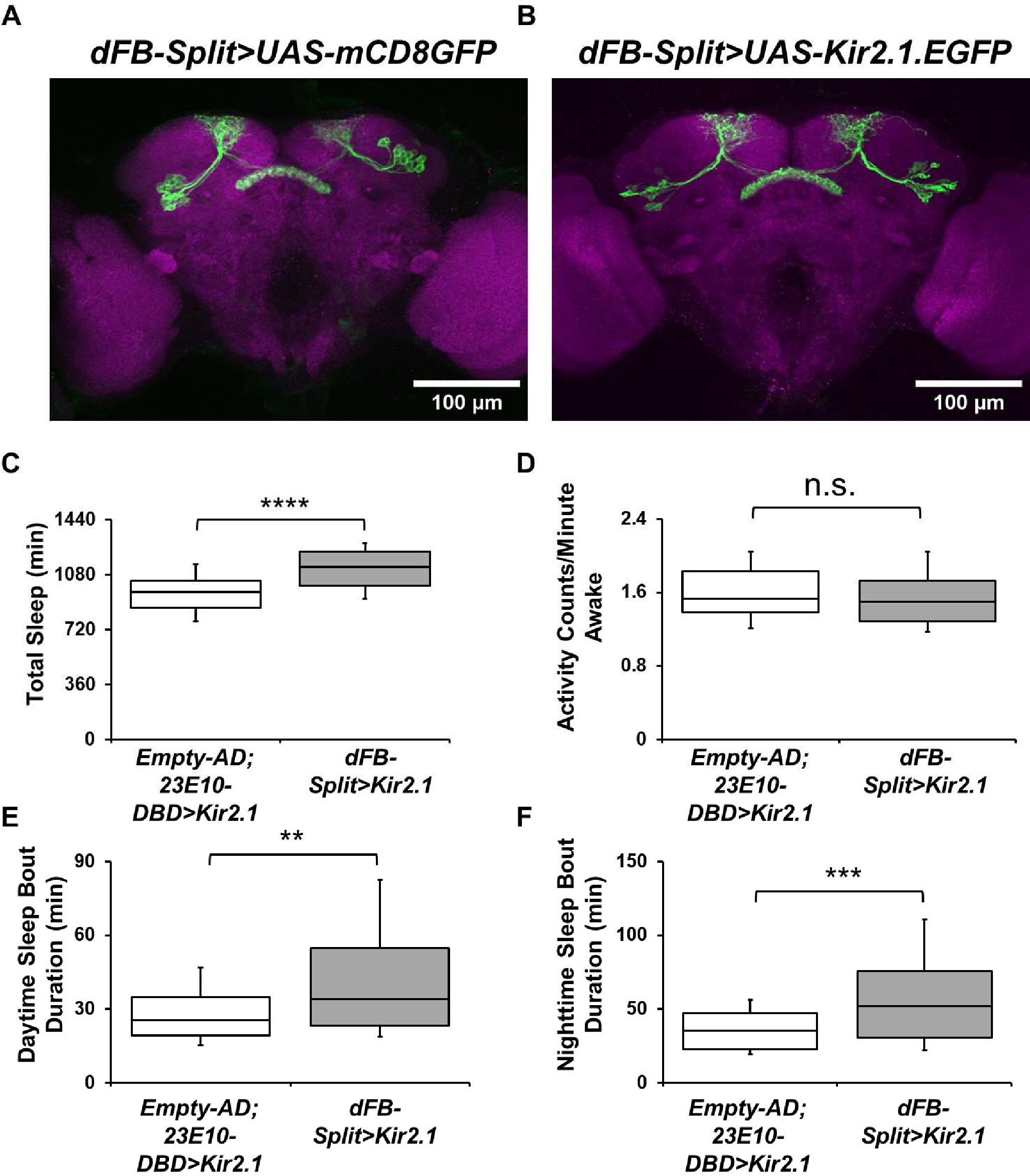

Supplement: S18 Fig — (A) Representative brain confocal stack of dFB-Split>UAS-mCD8GFP. Green, anti-GFP; magenta, anti-nc82. (B) Representative brain confocal stack of dFB-Split>UAS-Kir2.1.EGFP. We observed 23.40 ± 0.75 (n = 5) dFB23E10Ո84C10 neurons in dFB-Split>UAS-Kir2.1.EGFP brains. This number is similar to what we have observed for dFB-Split>UAS-mCD8GFP brains (Fig 1A). Green, anti-GFP; magenta, anti-nc82. (C) Box plots of total sleep (in minutes) for control and dFB-Split>Kir2.1 male flies. A two-tailed Mann–Whitney U test revealed that dFB-Split>Kir2.1 male flies sleep significantly more than controls. ****P < 0.0001. n = 69–75 flies per genotype. (D) Box plots of locomotor activity counts per minute awake for flies presented in C. A two-tailed Mann–Whitney U test revealed no differences between controls and dFB-Split>Kir2.1 male flies. n.s. = not significant. n = 69–75 flies per genotype. (E) Box plots of daytime sleep bout duration (in minutes) for flies presented in C. A two-tailed Mann–Whitney U test revealed that daytime sleep bout duration is increased in dFB-Split>Kir2.1 male flies. **P < 0.01. n = 69–75 flies per genotype. (F) Box plots of nighttime sleep bout duration (in minutes) for flies presented in C. A two-tailed Mann–Whitney U test revealed that nighttime sleep bout duration is increased in dFB-Split>Kir2.1 male flies. ***P < 0.001. n = 69–75 flies per genotype. The raw data underlying parts B, C, D, E, and F can be found in S1 Data. (TIF) [file pbio.3003014.s018.tif]

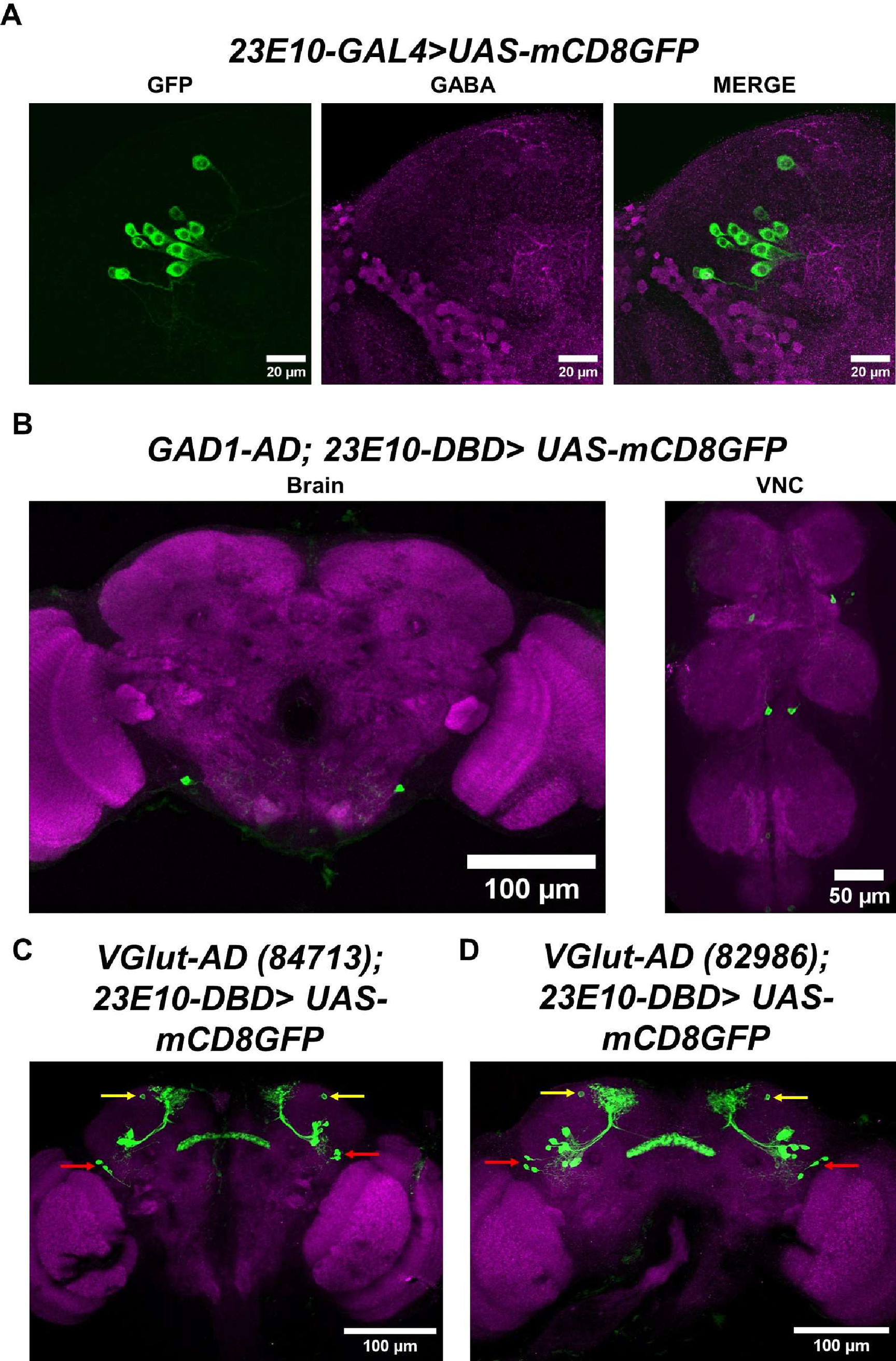

Supplement: S19 Fig — (A) Representative confocal stack of a female 23E10-GAL4>UAS-mCD8GFP brain stained with GFP and GABA antibodies and focusing on dFB cell bodies. Green, anti-GFP; magenta, anti-GABA. (B) Representative confocal stack of a female Gad1-AD; 23E10-DBD>UAS-mCD8GFP brain and VNC. Green, anti-GFP; magenta, anti-nc82 (neuropile marker). (C) Representative confocal stack of a female VGlut-AD (84713); 23E10-DBD>UAS-mCD8GFP brain. Yellow and red arrows show non-dFB neurons. Green, anti-GFP; magenta, anti-nc82 (neuropile marker). (D) Representative confocal stack of a female VGlut-AD (82986); 23E10-DBD>UAS-mCD8GFP brain. Yellow and red arrows show non-dFB neurons. Green, anti-GFP; magenta, anti-nc82 (neuropile marker). (TIF) [file pbio.3003014.s019.tif]

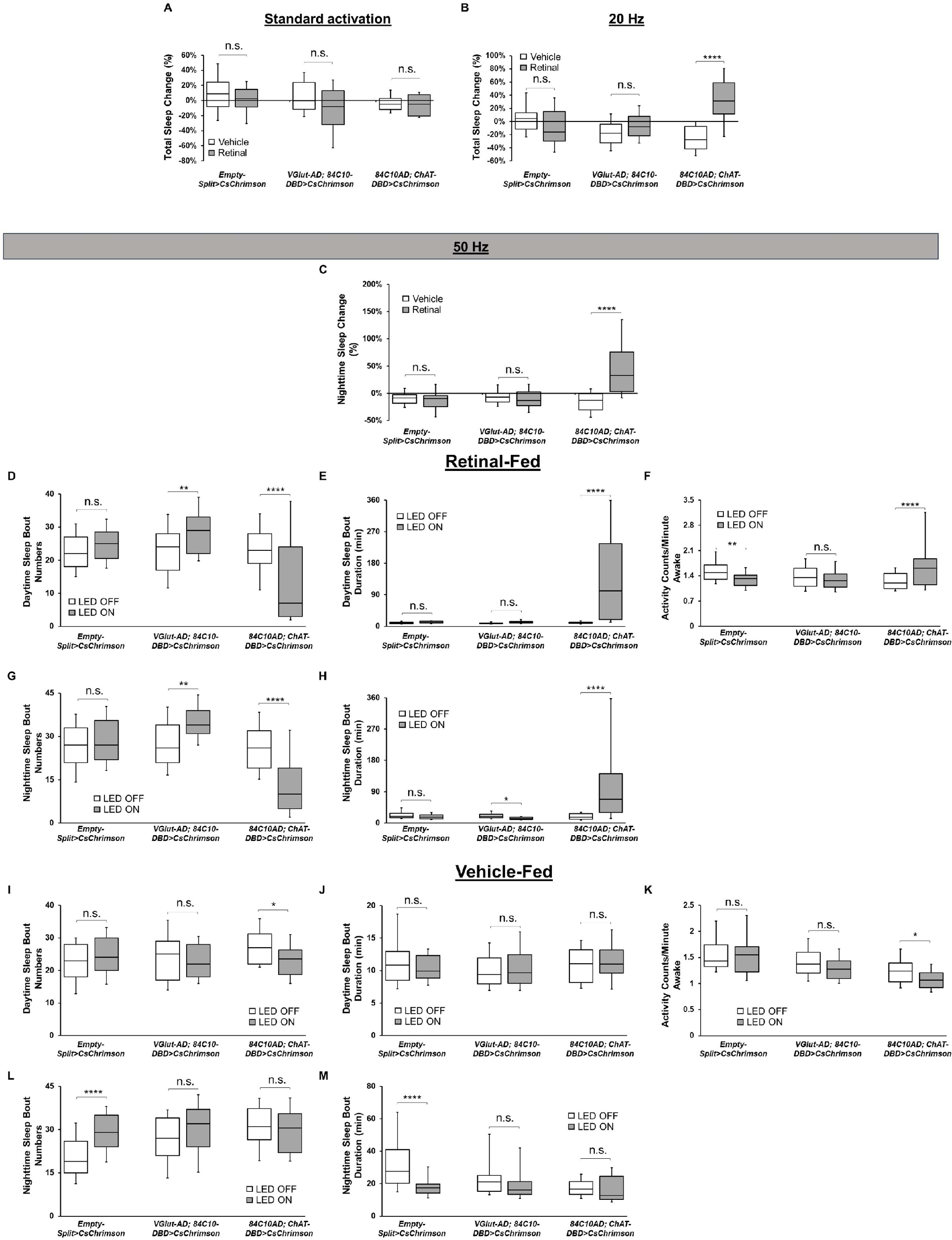

Supplement: S20 Fig — (A) Box plots of total sleep change in % for female control (Empty-Split), VGlut-AD; 84C10-DBD, and 84C10-AD; ChAT-DBD flies expressing CsChrimson under regular optogenetic activation (5 ms LED ON, 95 ms LED OFF, with a 4 s delay between pulses). Two-way ANOVA followed by Sidak’s multiple comparisons found no difference. n.s. = not significant. n = 12–36 flies per genotype and condition. (B) Box plots of total sleep change in % for female control (Empty-Split), VGlut-AD; 84C10-DBD, and 84C10-AD; ChAT-DBD flies expressing CsChrimson under 20 Hz activation. Two-way ANOVA followed by Sidak’s multiple comparisons found that total sleep is increased in 84C10-AD; ChAT-DBD>CsChrimson. n.s. = not significant, ****P < 0.0001. n = 19–26 flies per genotype and condition. (C) Box plots of nighttime sleep change in % ((nighttime sleep on activation day-nighttime sleep on baseline day/nighttime sleep on baseline day) × 100) for control (Empty-Split>CsChrimson), VGlut-AD; 84C10-DBD>CsChrimson and 84C10-AD; ChAT-DBD>CsChrimson female flies presented in Fig 4H–K. Two-way ANOVA followed by Sidak’s multiple comparisons revealed that activating 84C10-AD; ChAT-DBD neurons significantly increases nighttime sleep. n.s. = not significant, ****P < 0.0001. n = 32–47 flies per genotype and condition. (D) Box plots of daytime sleep bout numbers for retinal-fed flies presented in Fig 4K. Two-way repeated measures ANOVA followed by Sidak’s multiple comparisons test found that daytime sleep bout numbers are significantly increased in VGlut-AD; 84C10-DBD>CsChrimson and significantly decreased in 84C10-AD; ChAT-DBD>CsChrimson female flies that are stimulated with 627 nm LEDs. n.s. = not significant, **P < 0.01, ****P < 0.0001. n = 33–47 flies per genotype. (E) Box plots of daytime sleep bout duration for retinal-fed flies presented in Fig 4K. Two-way repeated measures ANOVA followed by Sidak’s multiple comparisons test found that daytime sleep bout duration is significantly increased in 84C10-AD; C [file pbio.3003014.s020.tif]

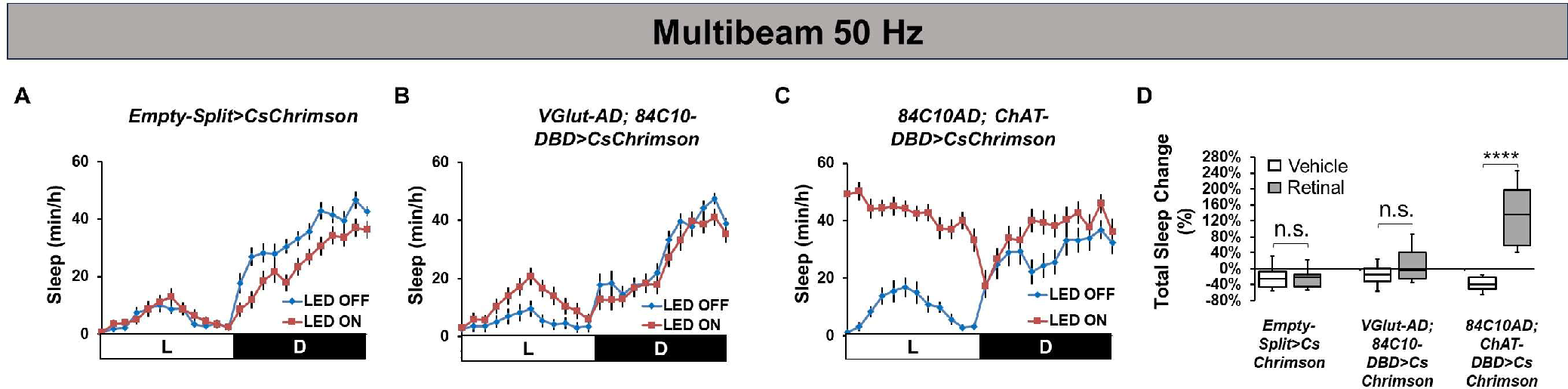

Supplement: S21 Fig — (A) Sleep profile in minutes of sleep per hour for day 2 (LED OFF, blue line) and day 3 (LED ON, red line) for retinal-fed Empty-Split control females expressing CsChrimson subjected to a 50 Hz optogenetic activation protocol (cycles of 5 ms LED ON, 15 ms LED OFF) obtained with the DAM5H multibeam system. (B) Sleep profile in minutes of sleep per hour for day 2 (LED OFF, blue line) and day 3 (LED ON, red line) for retinal-fed VGlut-AD; 84C10-DBD>CsChrimson female flies subjected to a 50 Hz optogenetic activation protocol (cycles of 5 ms LED ON, 15 ms LED OFF) obtained with the DAM5H multibeam system. (C) Sleep profile in minutes of sleep per hour for day 2 (LED OFF, blue line) and day 3 (LED ON, red line) for retinal-fed 84C10-AD; ChAT-DBD>CsChrimson female flies subjected to a 50 Hz optogenetic activation protocol (cycles of 5 ms LED ON, 15 ms LED OFF) obtained with the DAM5H multibeam system. (D) Box plots of total sleep change in % ((total sleep on activation day-total sleep on baseline day/total sleep on baseline day) × 100) obtained with the DAM5H system for control (Empty-Split>CsChrimson), VGlut-AD; 84C10-DBD>CsChrimson and 84C10-AD; ChAT-DBD>CsChrimson female flies under a 50 Hz optogenetic activation protocol. Two-way ANOVA followed by Sidak’s multiple comparisons revealed that activating 84C10-AD; ChAT-DBD neurons significantly increases total sleep. n.s. = not significant, ****P < 0.0001. n = 19–29 flies per genotype and condition. The raw data underlying part D can be found in S1 Data. (TIF) [file pbio.3003014.s021.tif]
