# Supplementary material for: The dorsal fan-shaped body is a neurochemically heterogeneous sleep-regulating center in Drosophila
Source: PLoS Biol. 2025 Mar 26;23(3):e3003014. doi: 10.1371/journal.pbio.3003014 (PMC12135941; doi:10.1371/journal.pbio.3003014)
Supplement: Attachment — Submitted filename: JonesDissel2024unpublished_review.pdf [file pbio.3003014.s035.pdf]

In this manuscript from Stephane Dissel's group, Jones et al present a very thorough investigation of the role of the dorsal fan-shaped body (dFSB) in sleep. This is a very important paper to current investigations of *Drosophila* sleep, and publication is highly recommended once the concerns outlined below are addressed.

Major revisions:

1. Given that previous studies have proposed that the dFSB is GABAergic, and that the 23E10∩84C10 split does not necessarily label all the dFSB neurons that 84C10 does, the authors should also perform an antibody stain of *23E10-Gal4>UAS-mCD8-GFP*, in case the dFSB neurons that are not part of the overlap are GABAergic.

2. To address the fact that constitutive KIR2.1 expression in the 23E10∩84C10 neurons increases sleep, the authors should use a temperature-sensitive repressor (tubGal80^{ts}) to silence the neurons during adulthood. *Shibire* is only effective at silencing chemical synapses, and Troup et al (*eLife* 2018) showed that the dFSB has gap-junction coupling (INX6); thus, using the temperature-sensitive repressor on KIR2.1 will address the possibility that loss of the gap junction coupling is necessary for sleep.

3. An alternative explanation for why constitutive KIR2.1 silencing increases sleep may be because of the imbalance created by silencing a population of heterogeneous neurons. To address this, the authors should test how using the cholinergic 84C10 split-Gal4 and glutamatergic 84C10 split-Gal4 alter sleep when used to drive KIR2.1. Only the csChrimson results of these drivers are shown.

Minor revisions:

1. In both the abstract (L44-46) and the introduction (L120-122), the authors refer to the recent De et al 2023 *Current Biology* paper, stating that De et al's conclusions were based on genetic tools that are not dFB-specific. While De et al do use 23E10-Gal4 to test homeostatic sleep, a more problematic issue with that study, leading to a discrepancy between their results and the ones presented by Jones et al in 2021 for an identical genetic manipulation, is their use of a very mild deprivation protocol. Mentioning the mild deprivation protocol is important for resolving an existing controversy in the literature.

2. Related to point #1, a major conclusion of this manuscript seems to be that the dFSB requires stronger stimulation in order to promote sleep than driver lines that includes VNC neurons, and this should be mentioned in the abstract.

3. In L164-165, the authors state, "This increase is likely explained by the need for a fly to perform tasks that are mutually exclusive to sleep in a reduced amount of waking time." – This sentence could be argued to be anthropomorphizing, and a more reasonable interpretation may be that a fly experiencing more restorative sleep has more energy for higher activity.

4. In Table S2, what is the difference between "X", "(-)", and "(+)"?

5. Include supplementary video comparing the flies doing different behaviors with and without csChrimson activation. Given the current concern in the field about whether neuronal activation causes sleep or some other form of immobility, as well as the trend towards characterizing micromovements and postural adjustments, sharing video documentation would be hugely beneficial to the field.

6. Fig 4C: Less confusing to say "GFP+ only" and "GFP+ total" and "ChAT+GFP" than to say "nothing".

7. Fig 4G: Having a blue square for Gad1-DBD, when there is no bar in the graph, is distracting and confusing.

-
