# Supplementary material for: The dorsal fan-shaped body is a neurochemically heterogeneous sleep-regulating center in Drosophila
Source: PLoS Biol. 2025 Mar 26;23(3):e3003014. doi: 10.1371/journal.pbio.3003014 (PMC12135941; doi:10.1371/journal.pbio.3003014)
Supplement: Attachment — Submitted filename: dissel_review.pdf [file pbio.3003014.s037.pdf]

The authors have clearly taken the reviewers' comments seriously, implementing a significant number of new experiments that enhance the manuscript's value. Notably, they added video analysis, as suggested by this reviewer and reviewer #1, conducted a thorough examination of the neurochemical nature of the cells in the 23E10 dFSB cluster, and incorporated a behavioral readout using STM to LTM consolidation.
The genetic manipulation within this work is of a very high standard, surpassing much of the field. Although there have been substantial improvements in the behavioral analysis, I remain unsure if it achieves the level of "unequivocality" it aims for and claims. This research marks an important advancement in the discussion of whether dFSB regulates sleep; however, it is not yet conclusive, in my opinion. The work holds considerable value, particularly in terms of reagents and quality standards. Nevertheless, I would advise caution in making bold claims, as this is unlikely to be the definitive conclusion. I have noted some weaknesses below that I believe impact the work.

Line 47-50: I suggest placing more emphasis on this point in the abstract to highlight its importance.

Line 60-75: If you need guidance on what content to remove, this paragraph could potentially be omitted. It doesn't contribute significantly and reads more like a review.

Line 131: As you did in the abstract, it's prudent to add a caveat regarding the necessity for much stronger activation of dFSB neurons. Additionally, the current data does not sufficiently support the claim that dFSB neurons are involved "especially in sleep homeostasis." Figures 1, 2, 4, and 5 primarily address baseline sleep, and the only main figure that covers sleep deprivation is Figure 3, which involves Kir2.1.

Line 333: I find the use of the term "standard" conditions problematic in this paper, both here and in other sections. Typically, a "standard" implies a broadly accepted protocol within the field, and there is no universally recognized standard for optogenetics. In this case, readers will not be familiar with your lab's specific "standard optogenetic protocol." It would be more effective to provide the precise quantitative and qualitative details instead.

Figure 2: I find the variability between treatments in this figure puzzling. Even when comparing the traces under "LED off" conditions, there's an unexpected degree of inconsistency. Notably, there are two overlapping sets: one between the blue traces in Standard (D), 20Hz (H), and 50Hz (J); and another between 10Hz (F) and 1Hz (B), which appears very different from the first set. I suspect this variability arises because one set of experiments included D, H, J, while another included B, F, with a single replicate for each. Although the number of flies (N) seems appropriate, it would be beneficial to provide more details on how often each experiment was replicated, especially since this is presented as the result that "unequivocally" resolves the issue.

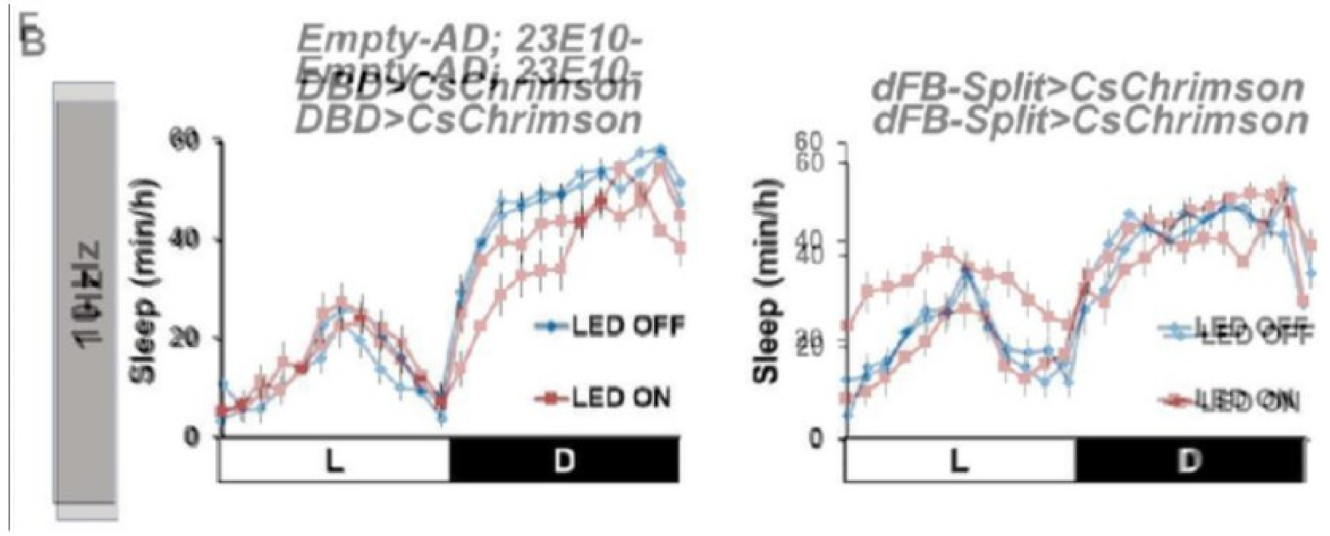

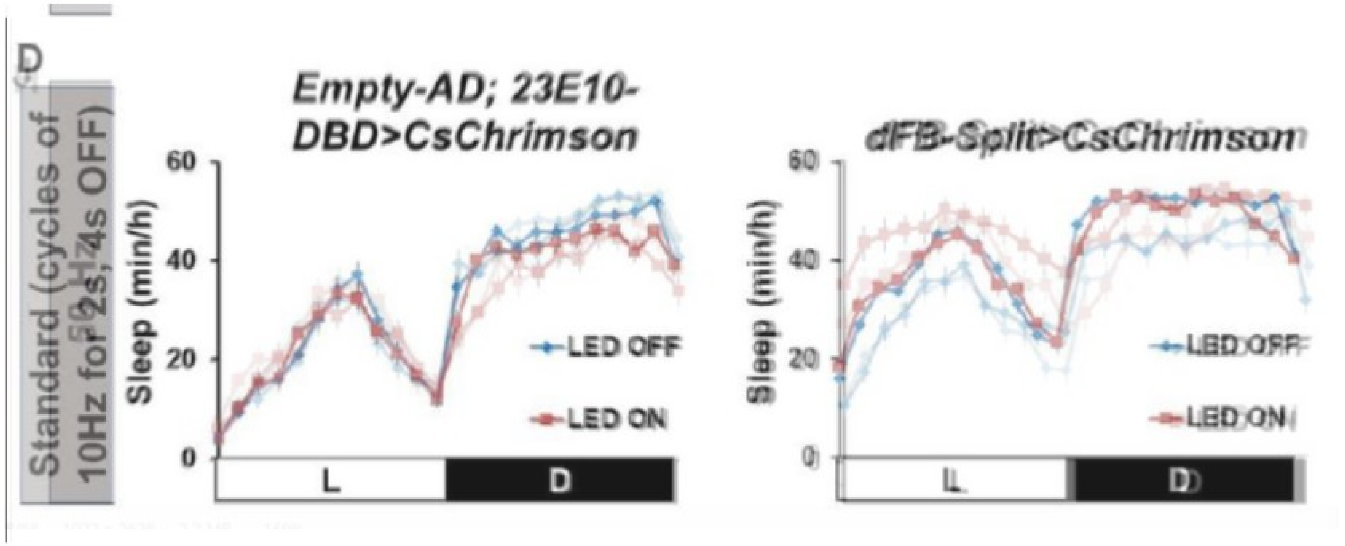

Line 372 – I think you need a reference if you want to claim that 31C is “more commonly used”. TrpA1 isoform B has an activation threshold of 27.8 ± 0.4 °C (Kang and Garrity, Nature 2012) so 29C is a commonly used activation temperature in behaviour. In fact, my feeling is that it may be even more commonly used in the sleep field exactly to avoid all those annoying confounding factors that derive from using 31C, as you correctly list at lines L174-178. As you correctly point out at L195 “it may be difficult to fully describe and characterize the sleep behaviors [...] when temperature itself has such a profound effect on sleep”.

Line 432 - “we conclude that activation of dFB23E10∩84C10 neurons increases sleep, rather than micromovements”. Please specify the activation conditions. Same for L441.

Line 443: The statement "we conclude that sufficient activation of dFB23E10∩84C10 neurons can increase sleep" may not be entirely accurate as worded. The use of "sufficient" suggests

that there is clear evidence that optogenetic stimulation below 10Hz or TrpA1 is not enough to activate firing, which may not be the case. It would be more appropriate to specify the conditions under which activation leads to increased sleep if such evidence is lacking. Consider revising this statement to accurately reflect the findings and limitations of the study.

L490 - “These results are somewhat surprising as they are similar to the ones obtained when activating dFB23E10∩84C10 neurons.” You may want to use more qualitative analysis than “similar”. Can you run a statistical comparison?

L499 – 527 As I stated in my first round of comments, the Occam’s razor possibility to explain this paradoxical effect is that sustained optogenetic activation phenocopies hyperpolarization through synaptic fatigue. There is a physiological limit to how fast and how long a neuron can fire and the fact that they can occasionally fire at 50Hz does not imply that the can sustain that rate for hours in a row (24hr in this case). The old Kandel papers showed that neurons show synaptic fatigue already at 1Hz. At that rate, sooner or later neurons do run out of vescicles and neurotransmitters.
This is admittedly not trivial to test because one would have to measure actual neuronal activity while at the same time stimulating the neuron. It can be done, but it would be too much for me to ask the authors to do this experiment. I would argue, though, that this caveat should be considered in the discussion.
