# Supplementary material for: The dorsal fan-shaped body is a neurochemically heterogeneous sleep-regulating center in Drosophila
Source: PLoS Biol. 2025 Mar 26;23(3):e3003014. doi: 10.1371/journal.pbio.3003014 (PMC12135941; doi:10.1371/journal.pbio.3003014)
Supplement: Attachment — Submitted filename: Response_to_Reviewers_auresp_3.docx [file pbio.3003014.s039.docx]

Reviewer #1: Jones et al (2024) did an excellent job addressing the issues that I raised with the previous version of their manuscript. I have one fairly major (but optional) suggestion, which is whether the organization of the paper would benefit from having Figure 3 (the long term memory data) moved to the end, so that characterization of the effect of silencing using the split-Gal4 (Fig 4 and 5) can follow the description of the effects of activating with the split-Gal4. Other than that, I have minor revision requests listed below.

We thank the Reviewer for their comments. We moved the LTM data right after the description of the dFB-Split GAL4 line in our resubmission to add support to the idea that the state that is induced when we activate dFB neurons is likely sleep since it supports LTM consolidation. This was done mostly because of Reviewer’s 3 comments. We have considered the Reviewer’s suggestion very carefully, and while there is merit to move the LTM data at the end of the manuscript, we still feel that a functional validation (memory consolidation) is needed as soon as possible in the manuscript to reinforce the message that dFB neurons promote sleep when activated.

Minor Revisions:

1. L152: Not sure, but I wonder if the authors meant to say "activator" not "actuator" here? I also feel like it should say, "cation channel" rather than "activator" to maintain a consistent/parallel structure with the TrpA1. Maybe to explicitly spell out what the channels are doing, L150-152 should say, "To activate the cells, each individual Split-GAL4" (not necessary for a field expert but would be beneficial if an undergrad or junior grad student were reading the paper).

We have replaced actuator with cation channel and adopted the suggestion made by the Reviewer.

2. L200-203: You don't describe the optogenetic approach here - I am assuming this is what you refer to as "our standard optogenetic protocol" in L333-334? From the response to the other reviewer, this seems to be the case, but explicitly mentioning it in the text the first time the optogenetic protocol is used will be helpful.

We have added this information in L200-203. Please note that we changed “standard” to “regular” in the manuscript and figures to satisfy Reviewer 3.

3. L333-334: "Next, we used our standard optogenetic protocol and again obtained no sleep increases when activating dFB23E10Ո84C10 neurons in females (Fig 2D and 2E)-." Remove the hyphen at the end; specify what is meant by "standard" here (It's shown in the figure but I think it's helpful to also have it explicitly spelled out in the text.)

We have removed the hyphen and added the information regarding the activation protocol.

4. Supplementary Movies 3-18 appeared to be missing from the submission?

These Movies are too big, so they are deposited here: <https://osf.io/64zh9/?view_only=d058f62c75254556a22f11bd28979287>

We have added this information in the manuscript.

5. L382 and Movie S3 and S4 caption: does "Cs", refer to "Canton-s" or "CsChrimson"?

Cs refers to the *Canton-S* wild-type stock, we have clarified this in the text and Movie captions.

Reviewer #2: I would like to thank the authors for addressing most of my concerns and adding a tremendous amount of new data to the manuscript. The new data presented in Figure S21 are really telling and it if there is a way to add them to the main manuscript that would be great. In my opinion, Figure 1 could be added to the supplementary figures, given that most of the lines tested have VNC-SP expression anyhow. That would make it possible to change Figure S21 to Figure 4. But I will leave it to the authors with respect to the decision they would like to make.

We thank the Reviewer for their comments and suggestions. We have moved Figure 1 to supplementary figures (now S1 Fig) and brought S21 Fig as a main Figure (Figure 5).

I accept the present manuscript provided that the authors address the minor concerns listed below:

-	S5-S18 movies: In the movie legend, no details with respect to the optogenetic stimulation can be found. I suggest to add the optogenetic stimulation protocol in the figure legend. This is important because, for example, in movie S18, I can see the flies are highly active during the stimulation phase. Is this a stimulation condition that is supposed to induce sleep? If so, this would significantly affect the conclusions of the paper and it should be addressed.

We employed a 20Hz optogenetic activation for these videos. We have added this information to the Movies legends. Movie S18 shows 4 *dFB-Split>UAS-CsChrimson* flies under LED treatment. Two of these flies (#8 and #9) are moving or performing micromovements for the entire 10-minutes duration. However, flies #6 and #7 are resting during the second part of the video (30% rest for #6 and 50% rest for #7). Note that both flies had 0% of rest under baseline conditions. This activation video shows the first 10 minutes after LED are turned on at 20Hz, immediately following the 10 minutes baseline video (recordings were done between 11am and 1pm). It is important to note that these videos are only 10 minutes snapshots during baseline and activation. Our analysis of these videos showed that, on average, *dFB-Split>UAS-CsChrimson* flies spend 0% ± 0% of the time resting during the 10-minutes baseline but spend 21% ± 7% of the time resting in the first 10 minutes of optogenetic activation. Our data shows that when analyzing a population of flies, activating dFB neurons increases rest, however, there is variability in how each individual fly respond in the first 10 minutes following the beginning of optogenetic activation. We have observed similar variability in individual fly responses when analyzing *dFB-Split>UAS-CsChrimson* flies activated at 20Hz with the MB system (Fig 1M), some flies in this experiment show rest in the first 10 minutes after LEDs are turned on, some do not. Note that for the data presented in Fig 1M, the amount of sleep in retinal fed flies in the first hour after the LEDs are turned on is 18.42 ± 6.33 min (vs 1.37 ± 0.76 min on baseline). These data indicate that flies do not sleep immediately upon optogenetic activation of dFB neurons. In addition, individual flies respond faster than others, probably reflecting individual differences in their internal state. For example, previous sleep history may modulate dopaminergic inhibition of dFB neurons. As a consequence, dFB cells may require different levels/duration of optogenetic activation to overcome this inhibition.

-	In their response to the reviewers, the authors state the following: 'As correctly stated by the Reviewer, our goal was to identify the contribution (if any) of 23E10-GAL4 dFB neurons in sleep regulation. Unfortunately, as demonstrated in S1 Fig and S1 Table, most 23E10-DBD containing Split-GAL4 FBS lines are not dFB-specific. More importantly, most of them contain the previously described sleep-promoting VNC-SP neurons. In addition, there is no 23E10-AD line available, so we are limited to using 23E10-DBD for creating Split-GAL4 tools.' In fact, there are two 23E10-AD lines available from Bloomington (Bloomington numbers 601444 and 601936). It is unclear to me as to why the authors have not performed these experiments, especially given how central this is for the logical flow of the manuscript. Did the authors test these lines? Are they not working?

The Reviewer is right, there is now two 23E10-AD lines available from the Bloomington stock center, but these have only been available very recently. Our studies have been initiated years ago, well before these two lines became available. We are not certain that at this stage, creating more Split-GAL4 lines using 23E10-AD would dramatically change/alter our conclusions. In addition, since 23E10-GAL4 expresses in many non-dFB neurons (Jones *et al.*, 2023), we are unsure whether Split-GAL4 lines created with 23E10-AD would be dFB-specific. As an example, we showed in this manuscript that two independent VGlut-AD; 23E10-DBD lines are not dFB-specific.

-	Figure 4R: Why do the authors use a temperature of 29°C as opposed of 31°C for sleep deprivation experiments?

We used 29° to mitigate the potential and reported effects of high temperature on sleep. We believe that these effects could be particularly problematic when assaying sleep homeostasis.

-	Lines 232-235: The authors state that '…, 7 are consistently increasing total sleep and sleep consolidation in females and males using both activation protocols'. In Figure 1, only 4 lines are consistently and significantly increasing total sleep using both activation protocols. Can the authors clarify what evidence their claim is based upon? I recommend to rephrase this sentence more precisely and also adjust the discussion accordingly (lines 728-730).

We apologize for the confusion. The Reviewer is right, 4 lines strongly increase total sleep using both thermogenetic and optogenetic in females. This number is 7 for males. We have changed the sentence to now read “Among these 16 lines, 4 are consistently increasing total sleep and sleep consolidation in females (FBS42, FBS45, FBS53, and FBS68) and 7 in males, using both activation protocols (FBS42, FBS45, FBS53, FBS68, FBS72, FBS81, and FBS84) (S2 Table). “

We also modified this description in the discussion.

-	Lines 315-316: The authors state '… our activation screens and anatomical analysis identified 16 sleep-promoting FBS lines expressing in diverse numbers of dFB neurons.' However, when looking at figure 1, only 4 lines are consistently and significantly increasing total sleep. I recommend to rephrase more precisely. Also, how can an anatomical screen identify sleep-promoting lines?

Again, we apologize for the confusion and have changed the sentence to now read “In conclusion, our activation screens identified 16 FBS lines, expressing in diverse numbers of dFB neurons, that modulate at least one sleep parameter (S2 Table).”

-	Lines 343-344: The authors state '… indicating that it is effective at activating neurons.' Given that different neurons show different physiological responses due to different biophysical/network properties, a given stimulation protocol that works in one neuronal type doesn't necessarily work in a different neuronal type. This is therefore not an argument that a protocol '.. is effective at activating neurons' in general. The protocols need to be adjusted to the type of neurons tested. I recommend that the authors rephrase this sentence.

We changed the sentence to now include “effective at activating some neurons”. Our goal was to say that this protocol has been working on some neurons, not that it works on all neurons, as the Reviewer correctly pointed.

-	Lines 502-503: The authors state 'As seen in S173A and S173B Fig, we observed no defects in dFB23E10Ո84C10 neuron numbers or processes when expressing Kir2.1, compared with controls'. This is difficult to conclude without proper quantification. I recommend to rephrase this sentence or add a quantification.

We agree with the Reviewer and have changed the sentence to now read “As seen in S18A and S18B Fig, we observed no gross morphological defects in dFB^23E10^**^Ո^**^84C10^ neuron numbers or processes when expressing Kir2.1, compared with controls.”

-	Line 514: The authors state '… without disrupting the anatomical properties of these cells'. I recommend to rephrase this sentence to account for the lack of quantification.

We changed the sentence to read “Thus, chronic hyperpolarization of dFB^23E10^**^Ո^**^84C10^ neurons increases sleep and sleep consolidation without disrupting the gross morphological properties of these cells.”

-	Lines 660-661: The authors state that 'Thus, these data suggest that … use another neuromodulator to promote sleep.' Another possible interpretation is that the remaining vGLUT transcripts (the knockdown being unlikely to be 100%) is sufficient to transmit downstream information. I recommend to rephrase this sentence unless the authors have some evidence for neuromodulators promoting sleep in these cells.

We thank the Reviewer for this suggestion. Yes, it is possible that there is enough remaining VGlut or VAChT even after we expressed RNAi against these transporters. It is also possible that either transporter can transmit downstream information, which could explain why knocking down only one of them with RNAi does not stop the sleep increase triggered by optogenetic activation. In addition, a recent preprint (Wolff *et al.*, 2024) demonstrates that the dFB expresses multiple neuropeptides. So, neuropeptides could also play a role. We have changed this section to now read “As seen in Fig 5D, we found that reducing VAChT or VGlut levels in dFB^ChAT^**^Ո^**^84C10^ neurons does not block the sleep increase observed when using a 50Hz optogenetic activation in female flies. There are multiple possible explanations for these findings. First, a subset of dFB^ChAT^**^Ո^**^84C10^ neurons also express glutamate (ChAT^+^, VGlut^+^ cells), so it is possible that these neurons use either glutamatergic or cholinergic transmission to downstream targets. This would explain why reducing levels of only one of these two single vesicular transporters cannot block the sleep increase obtained with optogenetic activation. Secondly, it is possible that there is enough vesicular transporter remaining in our RNAi experiments, enabling signaling to downstream targets. Alternatively, dFB^ChAT^**^Ո^**^84C10^ neurons may use another neuromodulator to promote sleep. Given that the central complex, which contains the dFB, is one of the most peptidergic area of the fly brain (60, 61), we think that it is extremely likely that dFB neurons are peptidergic. In fact, a recent preprint has identified many peptides expressed in the dFB (57).”

-	Lines 665-667: same comment than for lines 660-661

We have changed this sentence to now read “These data suggest that VGlut and VAChT may reduce the sleep-promoting capacity of dFB^VGlut^**^Ո^**^84C10^ neurons and that these cells may also use another neuromodulator to increase sleep when sufficiently activated.”

Typos:

-	Line 216: typo, 'S5A Fig' instead of 'S54A Fig'

-	Line 218: typo, 'S5B Fig' instead of 'S54B Fig'

-	Line 220: typo, 'S5C Fig' instead of 'S54C Fig'

-	Similar typos found in lines 221, 224

-	Line 488: 'S16A Fig.' instead of 'S164A Fig.'

-	Line 574: typo, 'Fig 5E and 5G' instead of 'Fig 54E and 54G'

-	Line 574: 'S20 Movies' instead of 'S204 Movies'

We are not sure what these issues are. We don’t see them in our manuscript.

Reviewer #3: The authors have clearly taken the reviewers' comments seriously, implementing a significant number of new experiments that enhance the manuscript's value. Notably, they added video analysis, as suggested by this reviewer and reviewer #1, conducted a thorough examination of the neurochemical nature of the cells in the 23E10 dFSB cluster, and incorporated a behavioral readout using STM to LTM consolidation.

The genetic manipulation within this work is of a very high standard, surpassing much of the field. Although there have been substantial improvements in the behavioral analysis, I remain unsure if it achieves the level of "unequivocality" it aims for and claims. This research marks an important advancement in the discussion of whether dFSB regulates sleep; however, it is not yet conclusive, in my opinion. The work holds considerable value, particularly in terms of reagents and quality standards. Nevertheless, I would advise caution in making bold claims, as this is unlikely to be the definitive conclusion. I have noted some weaknesses below that I believe impact the work.

We thank the Reviewer for their comments. We realize that the role of the dFB and sleep is hotly contested right now. We are also aware that one must be very aware about making bold claims. However, when taking the entirety of our results in consideration, using multiple dFB-specific lines, including the activation and silencing experiments, the RNAi data, and the consolidation of LTM, the easiest explanation for our data is that the dFB modulates sleep. Even when considering the neuronal fatigue possibility raised by the Reviewer, the fact remains that these manipulations change sleep. So, whether these phenotypes are due to activation or silencing of the neurons does not change the fact that sleep is altered. These data indicate that modulating the activity of dFB neurons changes sleep.

-------

Line 47-50: I suggest placing more emphasis on this point in the abstract to highlight its importance.

We have changed this sentence to now read “In this study, using a newly created dFB-specific genetic driver line, we demonstrate that optogenetic activation of the majority of 23E10-GAL4 dFB neurons promotes sleep and that these neurons are involved in sleep homeostasis.”

Line 60-75: If you need guidance on what content to remove, this paragraph could potentially be omitted. It doesn't contribute significantly and reads more like a review.

We think it is an important paragraph needed to introduce the necessity to use clean tools, as well as the complexity of such approaches in many systems.

Line 131: As you did in the abstract, it's prudent to add a caveat regarding the necessity for much stronger activation of dFSB neurons. Additionally, the current data does not sufficiently support the claim that dFSB neurons are involved "especially in sleep homeostasis." Figures 1, 2, 4, and 5 primarily address baseline sleep, and the only main figure that covers sleep deprivation is Figure 3, which involves Kir2.1.

We have changed the sentence in Line 131 to now read “Using this novel driver, we demonstrate that 23E10-GAL4 dFB cells regulate sleep and play a role in sleep homeostasis. Importantly, dFB neurons require relatively strong activation protocols to promote sleep, stronger than what we found for VNC-SP neurons (27). We also show that dFB-induced sleep promotes the consolidation of STM to LTM.”

We also want to point out that we performed homeostasis experiments with our neurotransmitter specific lines using Shi^ts1^, so that we have more than just the Kir2.1 data for homeostasis.

Line 333: I find the use of the term "standard" conditions problematic in this paper, both here and in other sections. Typically, a "standard" implies a broadly accepted protocol within the field, and there is no universally recognized standard for optogenetics. In this case, readers will not be familiar with your lab's specific "standard optogenetic protocol." It would be more effective to provide the precise quantitative and qualitative details instead.

Yes, this is our standard laboratory optogenetic protocol. We never intended to claim that this is a standard for the field. We have decided to change “standard” with “regular” throughout the manuscript and hope that this will satisfy the Reviewer.

Figure 2: I find the variability between treatments in this figure puzzling. Even when comparing the traces under "LED off" conditions, there's an unexpected degree of inconsistency. Notably, there are two overlapping sets: one between the blue traces in Standard (D), 20Hz (H), and 50Hz (J); and another between 10Hz (F) and 1Hz (B), which appears very different from the first set. I suspect this variability arises because one set of experiments included D, H, J, while another included B, F, with a single replicate for each. Although the number of flies (N) seems appropriate, it would be beneficial to provide more details on how often each experiment was replicated, especially since this is presented as the result that "unequivocally" resolves the issue.

We do not feel that these data are puzzling at all. Sleep is known to be notoriously sensitive to many internal and external factors. The Reviewer is right, the 1Hz and 10Hz experiments were performed for the revision of the original manuscript. The other activation protocols were done before. So, it is possible that for the 1Hz and 10Hz experiments, something was different (humidity, slight differences in food, etc….) and that this could induce variability from experiment to experiment. However, since we are measuring the difference between baseline and subsequent activation (within one experiment done with similar conditions), we think that these baseline differences are not an issue. Importantly, each of these experiments were replicated 2-6 times.

Line 372 - I think you need a reference if you want to claim that 31C is "more commonly used". TrpA1 isoform B has an activation threshold of 27.8 ± 0.4 °C (Kang and Garrity, Nature 2012 ) so 29C is a commonly used activation temperature in behaviour. In fact, my feeling is that it may be even more commonly used in the sleep field exactly to avoid all those annoying confounding factors that derive from using 31C, as you correctly list at lines L174-178. As you correctly point out at L195 "it may be difficult to fully describe and characterize the sleep behaviors [...] when temperature itself has such a profound effect on sleep".

While the activation threshold for TrpA1 is indeed 27.8 ± 0.4 °C, the paper cited by the Reviewer also show that with increasing temperature above the threshold, stronger activation can be achieved. It is thus fair to expect that TrpA1 will lead to stronger activation at 31°C than at 29°C. In fact, we showed during the revision for our previous PLOS Biology paper that we could not see any sleep increase when expressing TrpA1 in all 23E10-GAL4 neurons when using 29°. We only saw a sleep increase using 31°. However, we have chosen to change the sentence to now read “It is important to note that these conclusions were made using either a thermogenetic approach that may not be sufficient (29° instead of 31° for TrpA1 activation)”

Line 432 - "we conclude that activation of dFB23E10Ո84C10 neurons increases sleep, rather than micromovements". Please specify the activation conditions. Same for L441.

We used both 20Hz and constant LED activation for video analysis. We have changed the sentence in Line 432 to now read “Thus, based on our multibeam and video analysis, we conclude that either a 20Hz or a constant optogenetic activation of dFB^23E10^**^Ո^**^84C10^ neurons increases sleep, rather than micromovements.”

For arousal threshold, we used LED constantly ON. We have changed the sentence in Line 441 to now read “These data suggest that constant optogenetic activation of dFB^23E10^**^Ո^**^84C10^ neurons increases arousal threshold, an indicator of sleep depth.”

Line 443: The statement "we conclude that sufficient activation of dFB23E10Ո84C10 neurons can increase sleep" may not be entirely accurate as worded. The use of "sufficient" suggests that there is clear evidence that optogenetic stimulation below 10Hz or TrpA1 is not enough to activate firing, which may not be the case. It would be more appropriate to specify the conditions under which activation leads to increased sleep if such evidence is lacking. Consider revising this statement to accurately reflect the findings and limitations of the study.

We agree with the Reviewer and have changed the sentence to now read “Thus, we conclude that optogenetic activation of dFB^23E10^**^Ո^**^84C10^ neurons at 10Hz or above can increase sleep, sleep consolidation, and sleep depth.”

L490 - "These results are somewhat surprising as they are similar to the ones obtained when activating dFB23E10Ո84C10 neurons." You may want to use more qualitative analysis than "similar". Can you run a statistical comparison?

It was surprising to find that both optogenetic activation and chronic silencing of dFB^23E10^**^Ո^**^84C10^ neurons result in sleep increases. We have changed the sentence to now read “These results are somewhat surprising as both chronic silencing and optogenetic activation of dFB^23E10^**^Ո^**^84C10^ neurons result in sleep increases.”

L499 - 527 As I stated in my first round of comments, the Occam's razor possibility to explain this paradoxical effect is that sustained optogenetic activation phenocopies hyperpolarization through synaptic fatigue. There is a physiological limit to how fast and how long a neuron can fire and the fact that they can occasionally fire at 50Hz does not imply that the can sustain that rate for hours in a row (24hr in this case). The old Kandel papers showed that neurons show synaptic fatigue already at 1Hz. At that rate, sooner or later neurons do run out of vescicles and neurotransmitters.

This is admittedly not trivial to test because one would have to measure actual neuronal activity while at the same time stimulating the neuron. It can be done, but it would be too much for me to ask the authors to do this experiment. I would argue, though, that this caveat should be considered in the discussion.

While synaptic fatigue is possible when activating neurons for long periods of time, we think that this is not the case in our study. If 24h long optogenetic activation of both VGlut-AD; 84C10-DBD and 84C10-AD; ChAT-DBD neurons was leading to synaptic fatigue we would expect that these manipulations would lead to decrease sleep, because we showed that silencing these neurons with Shi^ts1^ decreases sleep. However, we agree that we should address this possibility and have added the following paragraph in our discussion “Since chronic silencing and optogenetic activation of dFB^23E10Ո84C10^ neurons both increase sleep, a potential issue is that our 24h-long optogenetic activation protocols lead to neuronal fatigue, which would phenocopy hyperpolarization. However, we feel that this is unlikely for multiple reasons. First, following 12h of sleep-deprivation, individual dFB neurons increase their firing frequencies, with some neurons capable of firing up to 50Hz (16). These data indicate that our optogenetic protocols, using 10Hz, 20Hz, and 50Hz frequencies to activate dFB neurons, are within the physiological range of normal firing properties for these cells. Second, when using neurotransmitter-specific dFB Split-GAL4 lines (see below), we show that activation of these dFB neurons promote sleep while acute silencing leads to wake. If sustained activation of these dFB neurons led to neuronal fatigue, we would expect to obtain the same behavioral effect as silencing, which is not the case. Rather, we explain the sleep increase seen when chronically hyperpolarizing all dFB^23E10Ո84C10^ neurons by a disruption of the balance between inhibitory and excitatory connections within the dFB.”
