# Supplementary material for: The dorsal fan-shaped body is a neurochemically heterogeneous sleep-regulating center in Drosophila
Source: PLoS Biol. 2025 Mar 26;23(3):e3003014. doi: 10.1371/journal.pbio.3003014 (PMC12135941; doi:10.1371/journal.pbio.3003014)
Supplement: Attachment — Submitted filename: Response to Reviewers.docx [file pbio.3003014.s038.docx]

**Response to Reviewers:**

Reviewer #1: In this manuscript from Stephane Dissel's group, Jones et al present a very thorough investigation of the role of the dorsal fan-shaped body (dFSB) in sleep. This is a very important paper to current investigations of Drosophila sleep, and publication is highly recommended once the concerns outlined below are addressed.

We thank the Reviewer for these comments.

Major revisions:

Given that previous studies have proposed that the dFSB is GABAergic, and that the 23E10∩84C10 split does not necessarily label all the dFSB neurons that 84C10 does, the authors should also perform an antibody stain of 23E10-Gal4>UAS-mCD8-GFP, in case the dFSB neurons that are not part of the overlap are GABAergic.

The Reviewer is right. Our dFB-Split line may not label all dFB neurons (including some 23E10-GAL4 expressing neurons). To address this, we performed anti-GABA staining in *23E10-GAL4>UAS-mCD8GFP* and found no GABA expression in any 23E10-GAL4 dFB neurons (S18A Fig). In addition, we have created and examined *GAD1-AD; 23E10-DBD>UAS-mCD8-GFP* flies and found no expression in the dFB (S18B Fig). Thus, we are confident in stating that the dFB is not GABAergic. Note that there was expression in other cells in the brain and VNC in this Split-GAL4 line, supporting the idea that there are non-dFB GABAergic neurons in 23E10-GAL4.

2. To address the fact that constitutive KIR2.1 expression in the 23E10∩84C10 neurons increases sleep, the authors should use a temperature-sensitive repressor (tubGal80ts) to silence the neurons during adulthood. Shibire is only effective at silencing chemical synapses, and Troup et al (eLife 2018) showed that the dFSB has gap-junction coupling (INX6); thus, using the temperature-sensitive repressor on KIR2.1 will address the possibility that loss of the gap junction coupling is necessary for sleep.

We acknowledge that the constitutive expression of Kir2.1 could create issues, especially when expressed during development. Unfortunately, the GAL80 repressor does not work on the Split-GAL4 system, so we cannot perform the experiment suggested by the Reviewer.

However, we provide new data in which we acutely silenced dFB neurons contained within VGlut-AD; 84C10-DBD and 84C10AD; ChAT-DBD using Shibire^ts1^ and show that these manipulations significantly reduce sleep (Fig 5M-P) and affect sleep homeostasis (Fig 5R).

We explain our data expressing Kir2.1 in 23E10∩84C10 neurons by the neurochemically heterogenous nature of these cells, expressing the main excitatory neurotransmitter in *Drosophila*, acetylcholine, as well as glutamate. A recent study (Hasenhuetl *et al*., 2024) provides evidence of strong inhibitory connections between individual dFB neurons and propose that glutamate is an inhibitory neurotransmitter in the dFB. It is thus possible that chronically silencing all dFB neurons may disrupt the balance of inhibitory and excitatory connections within the dFB, making the interpretation of genetic manipulations difficult to predict.

3. An alternative explanation for why constitutive KIR2.1 silencing increases sleep may be because of the imbalance created by silencing a population of heterogeneous neurons. To address this, the authors should test how using the cholinergic 84C10 split-Gal4 and glutamatergic 84C10 split-Gal4 alter sleep when used to drive KIR2.1. Only the csChrimson results of these drivers are shown.

We agree with the Reviewer that the constitutive expression of Kir2.1 in neurochemically heterogeneous 23E10∩84C10 neurons may disrupt the balance of inhibitory and excitatory connections within the dFB. As mentioned above, we have expressed Shibire^ts1^ in VGlut-AD; 84C10-DBD and 84C10AD; ChAT-DBD neurons and demonstrate that these manipulations significantly reduce sleep (Fig 5M-P). Since Shibire^ts1^ provides the opportunity to perform acute silencing, its use alleviates potential developmental issues created by the chronic expression of Kir2.1. The results obtained when manipulating VGlut-AD; 84C10-DBD and 84C10AD; ChAT-DBD dFB neurons are easier to understand, sleep promotion when activated and sleep loss when silenced.

Minor revisions:

1. In both the abstract (L44-46) and the introduction (L120-122), the authors refer to the recent De et al 2023 Current Biology paper, stating that De et al's conclusions were based on genetic tools that are not dFB-specific. While De et al do use 23E10-Gal4 to test homeostatic sleep, a more problematic issue with that study, leading to a discrepancy between their results and the ones presented by Jones et al in 2021 for an identical genetic manipulation, is their use of a very mild deprivation protocol. Mentioning the mild deprivation protocol is important for resolving an existing controversy in the literature.

The Reviewer is right, we have changed the abstract to now read “A recent publication replicated our finding that 23E10-GAL4 contains sleep-promoting neurons in the VNC. However, it also proposed that the dFB is not involved in sleep regulation at all. Importantly, this suggestion was made using genetic tools that are not dFB-specific and a very mild sleep deprivation protocol.” and the part of the introduction to “In fact, a recent study suggests that the dFB has no sleep-regulating capacity (28). However, the conclusions made by these authors are based on data obtained with non dFB-specific tools and a very mild sleep deprivation protocol.”

2. Related to point #1, a major conclusion of this manuscript seems to be that the dFSB requires stronger stimulation in order to promote sleep than driver lines that includes VNC neurons, and this should be mentioned in the abstract.

The Reviewer is correct, dFB neurons require stronger stimulation to promote sleep. We have modified the abstract to include “In this study, using a newly created dFB-specific genetic driver line, we demonstrate that the majority of 23E10-GAL4 dFB neurons can promote sleep when activated and that these neurons are involved in sleep homeostasis. We also show that dFB neurons require stronger stimulation than VNC-SP cells to promote sleep.”

3. In L164-165, the authors state, "This increase is likely explained by the need for a fly to perform tasks that are mutually exclusive to sleep in a reduced amount of waking time." - This sentence could be argued to be anthropomorphizing, and a more reasonable interpretation may be that a fly experiencing more restorative sleep has more energy for higher activity.

We have removed this sentence from the manuscript.

4. In Table S2, what is the difference between "X", "(-)", and "(+)"?

We added the following sentence in the legend of Table S2 “X indicates a significant increase between baseline and activation days, (+) indicates a significant increase compared with controls during thermogenetic activation, and (-) indicates a significant decrease.”

5. Include supplementary video comparing the flies doing different behaviors with and without csChrimson activation. Given the current concern in the field about whether neuronal activation causes sleep or some other form of immobility, as well as the trend towards characterizing micromovements and postural adjustments, sharing video documentation would be hugely beneficial to the field.

First, we validated the multibeam (MB) system by pairing MB analysis with video recording (S15 Fig, S3 and S4 Movies). Our data show that the MB is sensitive and accurate enough to detect sleep and micromovements. Then, we performed new MB+Video analysis to flies in which dFB^23E10^**^Ո^**^84C10^ neurons were activated at 20Hz. We have added videos of control and dFB-Split>CsChrimson flies before and during optogenetic activation in this resubmission (S5-S18 Movies).

6. Fig 4C: Less confusing to say "GFP+ only" and "GFP+ total" and "ChAT+GFP" than to say "nothing".

This is a good suggestion. We have changed the Figure accordingly. Note that now this is Fig 5C.

7. Fig 4G: Having a blue square for Gad1-DBD, when there is no bar in the graph, is distracting and confusing.

We agree with the Reviewer and have changed the Figure accordingly. It is now Fig 5G.

Reviewer #2: In the Drosophila brain, a group of dorsal fan-shaped body neurons marked by the 23E10-GAL4 driver line was found to be necessary and sufficient for promoting sleep. However, a recent study from the same lab revealed that the 23E10-GAL4 driver line also labels neurons outside of the brain, namely in the ventral nerve cord, which promote sleep when artificially activated. Consequently, behavioral results obtained with 23E10-GAL4 might be inconclusive, as the observed effects could be due to neurons in either the ventral nerve cord or in the dorsal fan-shaped body. To address this issue, the authors of this manuscript have identified driver lines that specifically label neurons in the dorsal fan-shaped body but not in the ventral nerve cord, demonstrating that activation of these neurons promotes sleep. This manuscript thus resolves a significant issue in the field of Drosophila sleep research. I recommend this manuscript for publication, provided that key points are addressed in a revised version.

We thank the Reviewer for these comments.

Key points:

*	Figure 3: Here, the empty-split > Kir 2.1 group sleeps abnormally little (especially when compared to the empty-split > CsChrimson). Thus, the sleep-promoting effect observed upon Kir2.1 expression using the dFB-Split line might not be a true effect but rather due to the control group. The authors should add an experiment in which they use proper parental controls, i.e., Kir2.1/+, empty-split/+, dFB-split/+, empty-split > Kir2.1, dFB-split > Kir2.1 in order to validate (or not) their conclusion.

We thank the Reviewer for this suggestion. It turns out that we ran all parental controls for these experiments but did not show them in our submission for clarity. We have now included these data in Fig 4A-D. The inclusion of all parental controls does not change our conclusion, expressing Kir2.1 in dFB^23E10^**^Ո^**^84C10^ neurons significantly increases sleep.

*	Figure 4: It is unclear to me as to why the authors use split-GAL4 combinations based on 84C10-AD instead of 23E10-DBD or 23E10-AD given that the initial goal of the paper was to understand cellular heterogeneity in 23E10-GAL4 (as for example written in lines 519-521 in the discussion). The authors should test the following split-GAL4 combinations in sleep experiments and also provide anatomical pictures: 23E10-AD/-DBD + Gad1-AD/-DBD and 23E10-AD/-DBD + ChAT-AD/-DBD and 23E10-AD/-DBD + Vglut-AD/-DBD.

As correctly stated by the Reviewer, our goal was to identify the contribution (if any) of 23E10-GAL4 dFB neurons in sleep regulation. Unfortunately, as demonstrated in S1 Fig and S1 Table, most 23E10-DBD containing Split-GAL4 FBS lines are not dFB-specific. More importantly, most of them contain the previously described sleep-promoting VNC-SP neurons. In addition, there is no 23E10-AD line available, so we are limited to using 23E10-DBD for creating Split-GAL4 tools. Since there is no ChAT-AD line available either, we cannot create 23E10-AD; ChAT-DBD or ChAT-AD; 23E10-DBD lines. However, we have created two VGlut-AD; 23E10-DBD lines (using the two different VGlut-AD lines available from the stock center). Both capture multiple dFB neurons, reinforcing the notion that many dFB neurons are glutamatergic. However, both lines also express in 8 neurons that are not dFB cells (S18C and S18D Fig, S23 and S24 Movies). Note that the non-dFB neurons labelled in both lines appear similar. We thus conclude that both our VGlut-AD; 23E10-DBD lines are not dFB-specific and cannot be used to study a role for the dFB in sleep regulation. We are aware of a preprint from the Miesenbock group (Hasenhuetl *et al*., 2024) that has used a VGlut-AD; 23E10-DBD line as a dFB-specific line. However, it is not clear which VGlut-AD line was used in this preprint. It is possible that this group has access to another VGlut-AD line, unknown to us, though we don’t think this is the case.

We are also providing in this resubmission pictures of a *Gad1-AD; 23E10-DBD>GFP* brain and VNC and show that there is no expression in the dFB, reinforcing our conclusion that the dFB is not GABAergic (S18B Fig).

In conclusion, we feel that our dFB-Split line (which contains 75%-85% of all 23E10-GAL4 dFB neurons), our VGlut-AD; 84C10-DBD and 84C10-AD; ChAT-DBD lines are the best tool at our disposal to investigate a role for the dFB in sleep regulation, because all 3 lines are dFB-specific.

*	Figure 4: Do the authors think that the release of Acetylcholine or Glutamate is needed for the observed sleep-promoting effect of VGlut-AD;84C10-DBD or ChAT-AD;84C10-DBD? The authors could knock down the respective vesicular transporters and check whether the sleep-promoting effect is still observed upon optogenetic stimulation.

This is a great suggestion by the Reviewer. We have now expressed RNAi for VGlut and VAChT in VGlut-AD; 84C10-DBD and ChAT-AD; 84C10-DBD neurons (S21A-S21C Fig). Our data show that for dFB^VGlut^**^Ո^**^84C10^ neurons, reducing levels of VGlut or VAChT (using two independent RNAi lines for each) significantly reduces sleep, suggesting that in these cells, cholinergic and glutamatergic transmission promote sleep. For dFB^ChAT^**^Ո^**^84C10^ neurons, we obtained the opposite result, as flies with reduced VGlut or VAChT levels (1 RNAi line for each) increased sleep. These data suggest that in dFB^ChAT^**^Ո^**^84C10^ neurons, both transporters promote wake.

We then reduced the expression levels of VGlut or VAChT while simultaneously activating dFB^VGlut^**^Ո^**^84C10^ or dFB^ChAT^**^Ո^**^84C10^ neurons (S21D Fig). Our data shows that reducing the levels of both transporters does not block the sleep-promoting effect of activation of dFB^ChAT^**^Ո^**^84C10^ neurons. These data indicate that these neurons rely on another neuromodulator, probably a neuropeptide, to promote sleep when activated. For dFB^VGlut^**^Ո^**^84C10^ neurons, reducing levels of VGlut or VAChT, increased the capacity of these cells to promote sleep when activated, again suggesting that another neuromodulator is responsible for this increase.

*	In general, I find the nomenclature for the split-GAL4 lines used (e.g., FBS6, VNC-Split, dFB-Split) confusing, non-informative and non-consistent throughout the figures of the paper (e.g. Empty-Split in Figure 3 versus Empty-AD;23E10-DBD in Figure 2, is this the same control line?). It would be helpful to change the names to the actual enhancer nomenclature throughout the paper to avoid any confusion for the reader.

We apologize for the confusion. Just to clarify, the control for all the FBS lines we screened, and the dFB-split line is Empty-AD; 23E10-DBD, as 23E10-DBD is a common element in all these lines. For the lines shown in Figure 5 (VGlut-AD; 84C10-DBD and 84C10AD; ChAT-DBD), the control is Empty-Split (Empty-AD; Empty-DBD). We have corrected all figures to ensure clarity about controls.

We also think that writing the full genotype of each line in each figure panel would make the figures extremely busy and difficult to read. This is why we chose the nomenclature we are using (detailed in S2 Table with AD component of each FBS line).

Minor points:

*	Figure 1C: please indicate what protocol was used for the optogenetic stimulation experiments, was it the standard protocol the authors refer to in Figure 2B? If so, could it be that some lines tested would actually show an effect given that the standard protocol used in Figure 2B is not effective for most non-bowtie-containing lines?

The activation protocol used in our screen is our standard protocol. We have added this information to Figure 1C. It is a protocol that is strong enough to activate VNC-SP neurons using a specific Split-GAL4 line we previously described (Jones *et al*., 2023). It is possible in principle that some FBS lines may show an effect using a stronger optogenetic activation. However, since most FBS lines express in VNC-SP neurons in addition to the dFB, it would be impossible to unequivocally assess the cellular origin of this sleep using these lines. There are only two FBS lines (FBS6 and FBS41) with no VNC-SP expression, both have a small number of dFB neurons, so these lines may not be optimal to assess a role for the dFB in sleep. We feel that our dFB-Split line that contains >80% of 23E10-GAL4 dFB neurons is the best tool to assess the contribution of these cells to sleep. In addition, our GFP-DD experiment suggest that it is the strength of expression within VNC-SP neurons that dictate whether a given FBS line will or won’t show a sleep increase upon neuronal activation.

*	Figure 2B: please indicate in grey box what 'Standard' refers to.

Our standard protocol is set to a pulse cycle of (5ms on, 95ms off) x 20 with a 4 second delay between pulse cycles. We added this information to the Figure (now Fig 2D).

*	Figure 2M/N: please indicate in figure legend what optogenetic stimulation protocol was used.

These experiments were done using LED on constantly. We have added this information to the Figures (now Fig 2Q and 2R).

*	Figure 4B: please provide higher-magnification pictures, it is very hard to see any colocalization.

We have included novel pictures for this figure (now Fig 5B).

Reviewer #3: This manuscript by Jones et al. adds to a saga of manuscripts investigating the role of the dFB in sleep regulation. The authors screen a combination of split GAL4 lines and show that the VNC component of 23E10 neurons likely accounts for the sleep phenotype observed under "standard" conditions of activation. The dFB-only component does not show any sleep phenotype under standard conditions, but some sleep phenotype does appear under "non-standard" manipulations (e.g., unusually high optogenetic activation and weirdly enough, in which chronic over-activation leads to the same phenotype as chronic inactivation).

We disagree with the Reviewer; we do not believe we are using “unusually high optogenetic activation”. It is important to note that a previous study done by the Miesenbock laboratory has investigated the firing properties of individual dFB neurons (Donlea *et al*., 2014). In this work, it was demonstrated that following a night of sleep deprivation, >65% of dFB neurons can fire up to 10Hz, >55% of dFB neurons can fire up to 20Hz and >20% of dFB neurons can fire up to 50Hz. In this resubmission, we have performed additional experiments using 1Hz and 10Hz optogenetic activation. We found that 1Hz activation is not sufficient to increase sleep in *dFB-Split>UAS-CsChrimson* flies (Fig 2B and 2C) but a 10Hz protocol significantly increases sleep (Fig 2F and 2G). Thus, our data demonstrate that an activation protocol of 10Hz or more increases sleep in *dFB-Split>UAS-CsChrimson* flies. Our activation protocols are well within the naturally occurring firing properties of individual dFB neurons. In addition, a recent preprint (Hasenhuetl *et al*., 2024), used a VGlut-AD; 23E10-DBD Split-GAL4 line to “specifically” (though in our hands this line is not dFB-specific, see S18C and S18D Fig) activate dFB neurons and found that an activation protocol consisting of pulse cycles of (3ms on, 47ms off) x 10 with a 0.5 second delay between pulse cycles significantly increased sleep. While the authors suggest that this activation protocol mimics slow-wave activity (SWA, 0.2-1Hz), we believe that it is in fact a protocol consisting of 20Hz activation for 0.5s followed by no activation for 0.5s. Thus, this activation protocol is comparable to the ones we are using.

Regarding the sleep increase obtained with chronic silencing of dFB^23E10Ո84C10^ neurons, we think that this is caused by **1)** the fact that dFB neurons are neurochemically heterogeneous, expressing both an excitatory (acetylcholine) and an inhibitory (glutamate) neurotransmitter (Hasenhuetl *et al*., 2024) and **2)** the strong levels of interconnection within the dFB (Hulse *et al.*, 2021; Hasenhuetl *et al*., 2024).

Using the neurotransmitter- and dFB-specific VGlut-AD; 84C10-DBD and 84C10-AD; ChAT-DBD lines, we find that acute activation of dFB neurons increases sleep (Fig 5H-5L) while acute silencing reduces sleep (Fig 5M-5P) and sleep homeostasis (Fig 5R).

The whole manuscript is rather verbose and would benefit from some synthetic editing. There is a lot of data being presented, mostly useful and relevant but not always so (especially in the supplementary figures). The genetic methodology employed is very careful and commendable. However, the physiological and behavioral analysis is less so and often employs self-made solutions that reinvent the wheel and lack necessary validation (for instance, the video analysis or the analysis of arousal threshold) or description (no code or detailed description of the apparatus is provided, nor validation).

Indeed, this study contains a lot of data, which we feel is necessary given the ongoing dispute about the role of the dFB in sleep. Unfortunately, or fortunately, this resubmission contains even more data.

We thank the Reviewer for his comment about our genetic approach. However, we respectfully disagree with the Reviewer when it comes to us reinventing the wheel. We are using state of the art behavioral approaches that have been validated and used by dozens of laboratories. It seems like the Reviewer is not convinced that the Trikinetics single beam and multibeam (MB) systems are sufficient to accurately describe sleep. We provide in this resubmission, evidence that the MB is accurate and sensitive enough in capturing multiple behaviors when comparing with video analysis (S15 Fig). Finally, our arousal protocol is similar to what has been done by many other laboratories. We subject sleeping flies to mechanical stimulus of increasing strength and measure the animal behavioral responses. We now provide the code for our arousal threshold in this resubmission.

I find the work suggestive but inconclusive, at least for a manuscript that clearly aspires to close the discussion once and for all. I think the authors are going in the right direction but, frankly, looking at the results, I am not reaching the same conclusion that the authors are putting forward. To me, the role of the dFB component of 23E10 appears weaker and weaker given that it is visible only under extreme conditions of manipulation.

We don’t agree that our activation protocols are extreme. Previous studies have demonstrated that individual dFB neurons can fire up to 50Hz following a night of sleep deprivation (Donlea *et al.*, 2014). In addition, we have performed additional experiments showing that *dFB-Split>UAS-CsChrimson* flies significantly increase sleep when stimulated at 10Hz or above. Thus, our activation protocols are well within the naturally occurring firing properties of individual dFB neurons. In addition, acute silencing of

dFB^VGlut^**^Ո^**^84C10^ or dFB^ChAT^**^Ո^**^84C10^ neurons, which decreases sleep (Fig 5M-5P) and blocks sleep homeostasis (Fig 5R), as well as the sleep phenotypes observed when reducing VGlut and VAChT levels (S21 Fig) in these neurons, support the conclusion that dFB neurons regulate sleep.

Here are some more specific comments:

**Figure 1**: Please find a way to combine the results from 1B and 1C either in the same graph or at least in a way that makes it visually easier to compare the same lines across the two different treatments (e.g., same width of the figure and just one panel on top of the other).

We have increased the width of Figure 1B to make comparisons with data in Figure 1C easier.

**Supplementary Figures 1-6**: The supplementary figures related to the dataset in Figure 1 are a bit sparse. For instance, the classical sleep pattern is shown only for the thermogenetics dataset (S1) but not for the optogenetics dataset. This is important because almost all your subsequent experiments rely on optogenetics, and it's impossible for the reader to compare the results between Figure 1 and, say, Figure 2 or Figure 4.

We have included all sleep patterns for the optogenetic screen in S4 Fig.

**Figure 2B**: What does "Standard" mean? The legend says "5ms LED ON, 95ms LED OFF, with a 4-second delay between pulses," but it's not immediately clear how this compares with the other stimulations used, because both 20Hz and 50Hz are indicated as having 5ms ON and 45ms OFF. I suspect that this is a typo and 50Hz is actually 5ms ON, 15ms OFF. I also assume that the 4-second delay still applies to all three conditions. Perhaps a schematic of the pulse protocol would help.

We apologize for our mistake, we have now corrected the legend for Figure 2J. 50Hz activation is indeed 5ms ON, 15ms OFF. There is no delay for the 20Hz and 50Hz activation protocols, only the standard activation protocol has a 4s delay.

The actual details of the optogenetics protocol used are very important and need to be clarified. I have a growing suspicion that this strong and prolonged activation simply leads to firing fatigue, and that is why your activation and Kir2.1 inactivation phenotypes are identical, both showing increased sleep. This would also explain why you do not see any sleep phenotype with ShiTS. Whichever the mechanism, the fundamental problem with a phenotype observed only when pushing the system beyond commonly adopted limits is that one starts losing control of what is actually going on. For instance, one could argue that some Chrimson is still expressed in the ventral nerve cord, not enough to be detectable through immunofluorescence or to be activated by standard regimes, but just enough to show a phenotype when the system is pushed beyond the limits. The joiner lab used tsh-GAL80 in their analysis and perhaps is worth considering using that too (and, by the way, it is unfair to say that those are "non-specific tools" - they are as specific as any other intersectional approach).

We agree that the activation protocol used in any optogenetics experiments matters and should be well described. However, we disagree that our activation protocols are extreme. As previously mentioned, individual dFB neurons can fire up to 50Hz. In addition, we have added milder activation protocols in this resubmission and show that 10Hz is sufficient to significantly increase sleep in *dFB-Split>UAS-CsChrimson* flies. We think that the sleep increase seen in *dFB-Split>UAS-Kir2.1* flies may be due to constitutive silencing of a heterogeneous group of dFB neurons. When taking into consideration the recent preprint from the Miesenbock lab (Hasenhuetl *et al*., 2024) that provides evidence of strong inhibitory connections between individual dFB neurons, and the neurochemical heterogeneity of dFB neurons expressing both an inhibitory and excitatory neurotransmitter, it is possible that silencing all dFB neurons may disrupt the balance of inhibitory and excitatory connections within the dFB, making the interpretation of genetic manipulations difficult to predict.

However, we show in this resubmission that acute silencing of glutamatergic and cholinergic neurons within the dFB significantly reduces sleep.

Is the Reviewer suggesting that our dFB-Split line retains some weak expression in the VNC-SP cells? Our confocal imaging analysis suggests that this is not the case. Unfortunately, the GAL80 repressor does not work on Split-GAL4 lines so we cannot do the experiment suggested by the Reviewer (*dFB-Split>UAS-Chrimson; tsh-GAL80*).

Importantly, we stand by our comment, 23E10-GAL4; tsh-GAL80 is not a dFB-specific tool, see below. There are clearly non-dFB neurons (yellow arrows) still expressing in 23E10-GAL4; tsh-GAL80 brains. Therefore, any behavioral effects obtained with this line cannot be unequivocally assigned to dFB neurons. In conclusion, this tool cannot be used to specifically manipulate dFB neurons, contrary to the Split-GAL4 lines we created.

**Line 362, Figure 2MN**: "the DAM5H multibeam system, ruling out the possibility that we misregistered micromovements as sleep." I am afraid the multibeam system cannot rule out the possibility that you misregistered micromovements as sleep. DAM5H has 15 beams for a tube length of about 65 mm and therefore a resolution of 65/15 = 4.3mm. That is larger than the entire body of the animal so there is no guarantee that micromovements like grooming or seizures will be detected. There are now multiple studies that employ validated video tracking to show that 23E10 or 104y activation result in an increase of micromovements and/or seizures, and this is a critical aspect for a manuscript that aspires to set the record straight. Your video paradigm relies on a poorly described "manual scoring" while much more objective tools are now available diffusely employed in the literature.

There is a lot to address in this section. The comments made by this reviewer on the DAM5H multibeam (MB) system are incorrect on multiple aspects.

1) the distance between beams is 3mm and not 4.3mm, as wrongly stated by the Reviewer. This is demonstrated by a screenshot taken from the Trikinetics DAM5H technical sheet, and by a picture taken of one of the monitors we use in my laboratory (see below). While the calculation made by the Reviewer is correct, 65/15= 4.3mm, it seems that the Reviewer forgot that there is food at one end of the tube, so that the fly does not die of starvation during the experiment. The fly does not have access to the 65mm of tube length. Thus, a fly can be tracked continuously by the MB system. In fact, we have never failed to track the position or behavior of a fly in the MB system.

2) The Reviewer suggests that the DAM5H MB system may not be sensitive enough to detect micromovements like feeding or grooming. We have conducted validation experiments, coupling MB behavioral analysis with video analysis and found that the MB is >90% accurate in picking micromovements (S15C Fig). The only small movements that the MB struggles to detect are proboscis extension, which has been associated with sleep in two studies (Van Alphen *et al.*, 2021, Keles *et al.*, 2024), a small postural change (dip of the abdomen), and single leg movement, both of which have also been associated with sleep (Keles *et al.*, 2024). Thus, the DAM5H has the sensitivity and accuracy to detect micromovements and does not falsely classify these as sleep.
3) There are a couple of studies that have suggested that activation of all 23E10-GAL4 neurons does not augment sleep but rather increases in-place movements. One of these studies is a preprint employing 1Hz optogenetic activation for 5 minutes of *23E10-GAL4>UAS-CsChrimson* flies that are housed in a very small chamber (to allow for high resolution video analysis) (Keles *et al.*, 2024). The authors suggest that these manipulations lead to an increase in grooming and in-place movements. Based on this, the author concluded that activation of 23E10-GAL4 neurons does not induce sleep. When looking at the video provided in this study (Keles *et al.*, 2024), it appears to us that the flies seek to walk but cannot because of the constrained environment. We show in this resubmission that 24h of 1Hz optogenetic activation of dFB^23E10Ո84C10^ neurons does not increase sleep (Fig 2B and 2C). So effectively, we agree with the Keles preprint, 1Hz activation is not sufficient for dFB neurons to increase sleep. However, jumping from this conclusion to “the dFB does not promote sleep at all” is preposterous. Simply put, a 1Hz optogenetic activation is insufficient to activate dFB neurons, whether you perform video, single beam, or multibeam analysis.
4) We show that the state induced by activation of dFB^23E10Ո84C10^ neurons can consolidate LTM (Fig 3). LTM consolidation is one of sleep’s proposed functions. Would an increase in micromovements support LTM consolidation? We are not aware of such capacities. Thus, when considering the overwhelming evidence we are providing, we feel that the only conclusion is that activation of dFB^23E10Ո84C10^ neurons promotes sleep.

**Line 401**: "Acute silencing of dFB23E10Ո84C10 neurons has no effect on sleep [and this] further demonstrates that dFB23E10Ո84C10 neurons regulate sleep." I absolutely have no idea why you came to this conclusion. Silencing these neurons with ShiTS shows absolutely no effect, and yet you take this as confirmation that those neurons regulate sleep? I honestly don't get it.

We apologize for the confusion, yes, acute silencing of dFB23E10Ո84C10 neurons has no effect on sleep. We changed the sentence. Importantly, we now have data showing that expressing Shits1 using VGlut-AD; 84C10-DBD and 84C10-AD; ChAT-DBD significantly reduces sleep.

**Figure 3JK**: This experiment is important but complicated by the way it is conducted. Would you be able to see any effect on homeostasis using transient rather than developmental manipulation? Is there any homeostatic effect at all using TrpA1 with dFB23E10Ո84C10? This experiment is important and deserves more care because the entire literature assumes dFB is modulating homeostasis (the somnostat), but you dedicate to this aspect of the phenotype only this one experiment, which is inherently complicated by the way it's conducted.

We do not understand what’s complicated about this experiment. This is a protocol that has been employed in dozens of publications. Flies are mechanically sleep deprived for 12h at night and recovery sleep is measured over the subsequent 48h.

In addition, we have performed homeostasis experiments using acute silencing of dFB^VGlut^**^Ո^**^84C10^ or dFB^ChAT^**^Ո^**^84C10^ neurons (Fig 5R) and show that blocking the activity of cholinergic dFB neurons only during the recovery period significantly impairs sleep homeostasis. These new data confirm that dFB neurons regulate sleep, especially sleep homeostasis.
